# Anti-Androgenic Potential of Plant Extracts: Molecular Mechanisms, Synergistic Effects, and Nutritional Interventions

**DOI:** 10.3390/molecules31162849

**Published:** 2026-08-14

**Authors:** Yijing Yang, Yong Pang, Jie Zhang, Li Ren

**Affiliations:** College of Food Science and Engineering, Jilin University, Changchun 130062, China

**Keywords:** anti-androgenic effects, androgen receptor, androgen-related disorders, plant extracts, mixture effects

## Abstract

Androgen dysregulation impairs the homeostasis of the prostate, hair follicles, and ovaries, driving the pathogenesis of prostate cancer (PCa), benign prostatic hyperplasia (BPH), androgenetic alopecia (AGA), and polycystic ovary syndrome (PCOS). Current antiandrogen therapies are constrained by drug resistance, off-target toxicity, and limited long-term efficacy. Plant extracts, enriched with bioactive constituents such as polyphenols, alkaloids, and flavonoids, represent promising multi-target candidates. Therefore, focusing on plant extracts—particularly those of dietary origin—this review summarized the mechanisms by which they regulate androgen-dependent and androgen-independent signaling pathways, and recent advances in nutritional interventions targeting androgen-responsive organs. Furthermore, it discussed the multi-component combined effects and factors affecting in vivo exposure of plant extracts, including metabolic transformation and tissue distribution, which may influence the anti-androgenic activity. In summary, anti-androgenic plant extracts primarily target androgen synthesis and AR signaling across various androgen disorder models, with crosstalk into cell growth and cycle, anti-inflammatory pathways, and regulation of growth factors. This multi-target regulation relies on the mixture effects of plant extracts. Future research should focus on three directions: extract standardization, clinical validation, and advanced delivery systems.

## 1. Introduction

As critical chemical messengers involved in physiological regulation, the hormonal system comprises hydrophilic peptide and protein hormones, amino acid derivatives, and the structurally distinct superfamily of steroid hormones [1,2,3]. Among steroid hormones, androgens are derived from cholesterol and share a conserved tetracyclic structure and nuclear receptor-mediated mechanism of action [4]. Androgen signaling plays essential roles in reproductive development, secondary sexual characteristics, skeletal muscle growth, and erythropoiesis [5,6,7].

As a complex and critical endocrine regulatory system, androgen signaling is generally finely driven by the hypothalamic–pituitary–gonadal axis [8]. However, abnormal androgen signaling caused by altered receptor sensitivity or dysregulated metabolic enzyme activity may disrupt physiological homeostasis. Circulating testosterone is converted into the higher-affinity dihydrotestosterone (DHT) [9], then subsequently recruits and activates the nuclear androgen receptor (AR) to trigger the aberrant transcription of downstream genes. During this pathological process, uncontrolled cell proliferation, aberrant tissue remodeling, and inhibited apoptosis are cascaded [10]. Persistent dysregulation of androgen signaling can disrupt tissue homeostasis and contribute to pathological changes in androgen-responsive tissues.

Aberrant androgen signaling is closely associated with several androgen-dependent disorders, including prostate cancer (PCa) [11], Benign prostatic hyperplasia (BPH) [12], androgenetic alopecia (AGA) [13,14,15], and polycystic ovary syndrome (PCOS) [16], which impose substantial health burdens through tumor progression, urinary dysfunction, psychological distress, and metabolic and reproductive complications. Clinical strategies targeting the androgen-dependent disorders generally currently focus on androgen synthesis inhibition and signaling blockade. The commonly synthetic agents include 5α-reductase inhibitors [17] such as finasteride and dutasteride, and AR antagonists [18], including flutamide, bicalutamide, and spironolactone. Although effective, their long-term use is associated with adverse effects, including sexual dysfunction, ejaculatory disorders, gynecomastia, and post-finasteride syndrome characterized by depression and anxiety [19]. Moreover, antiandrogen therapy for PCOS may cause potential risks such as teratogenicity and menstrual irregularities [20]. These limitations have compelled researchers and clinicians to urgently explore safer, nutrition-oriented approaches suitable for long-term management. 

In this context, bioactive constituents derived from dietary plants have gradually emerged as a critical area of the intersection of nutrition and pharmacology [21]. Their structural diversity, multi-target mechanisms, and relatively favorable safety profiles have promoted increasing interest in anti-androgenic plant extracts. Epidemiological studies have suggested associations between Mediterranean or soy-rich Asian diets and reduced incidence of prostate diseases [22,23], supporting the potential role of dietary components in androgen regulation. Increasing evidence suggests the anti-androgenic potential of specific dietary extracts. For example, *Serenoa repens* [24], *Wedelia chinensis* [25], *Glycyrrhiza uralensis* Fischer [26], and *Garcinia cambogia* [27] crude extracts have been reported to regulate androgen-related pathways in various androgen-dependent disorder models. However, current reviews have mainly focused on specific diseases, individual compounds, or particular androgen-related targets. This highlights the need to an integrated perspective that considers plant extracts as multi-component systems across diverse androgen-dependent disorders.

Herein, we aimed to review the recent advances in anti-androgenic plant extracts, with a particular focus on dietary-derived sources. We first discuss how plant extracts regulate androgen signaling pathways, focusing on 5α-reductase inhibition, AR signaling blockade, and pathway crosstalk. Furthermore, this review summarizes the potential applications of plant extracts in PCa, BPH, AGA, and PCOS models. On this basis, attention is given to the mixture effects and spatiotemporal dynamics of plant extracts. Finally, the current limitations and future research directions are discussed. This review highlights the potential value of plant extracts as natural approaches for the regulation of androgen-related disorders and provides perspectives for their future development in functional foods, dietary supplements, and botanical therapeutics.

## 2. Methodology

Relevant literature was identified through searches of PubMed, Web of Science, Google Scholar, and Scopus databases using combinations of keywords including “plant extracts”, “anti-androgenic activity”, “androgen receptor”, “5α-reductase”, “prostate cancer”, “benign prostatic hyperplasia”, “androgenetic alopecia”, and “polycystic ovary syndrome”. A total of 691 records were identified, including 209 from PubMed, 184 from Web of Science, 226 from Google Scholar, and 72 from Scopus. Relevant literature published primarily between 2010 and 2025 was considered. 

After removal of 146 duplicate records, 542 records underwent title and abstract screening. The remaining 281 articles were then evaluated in full text according to the predefined inclusion and exclusion criteria, resulting in the exclusion of 72 articles. Finally, 212 references were included in this narrative review.

Studies were included if they:

(1) Investigated plant-derived materials, including extracts, isolated compounds, formulations, dietary materials, or fungal preparations;
(2) Evaluated anti-androgenic activity in relevant models;
(3) Reported measurable outcomes related to androgen regulation, molecular mechanisms, disease phenotypes, or translational aspects.

Studies were excluded if they:

(1) Lacked original experimental data;
(2) Did not investigate plant-derived materials or their related bioactive constituents;
(3) Focused on biological effects unrelated to androgen regulation or androgen-associated disorders.

Due to the narrative nature of this review, no formal meta-analysis or risk-of-bias assessment was performed.

## 3. Anti-Androgenic Effects of Plant Extracts

Androgens are a prominent class of steroid hormones, including testosterone, DHT, androstenedione, and dehydroepiandrosterone (DHEA) [28]. Physiological effects of androgens are largely mediated through the AR signaling cascade [29]. Over the past decade, anti-androgenic activities have been documented in various plant extracts, both in vitro and in vivo. Owing to their multi-active constituents, these extracts have been demonstrated to modulate one or more pathways targeting androgen-related imbalance, such as AR signaling and rate-limiting enzymes of androgen synthesis. In this context, elucidating the network mechanisms of this multi-target regulation is essential to understand how plant extracts amplify anti-androgenic efficacy.

### 3.1. Modulation of AR Signaling Cascades by Plant Extracts

**Figure 1:**
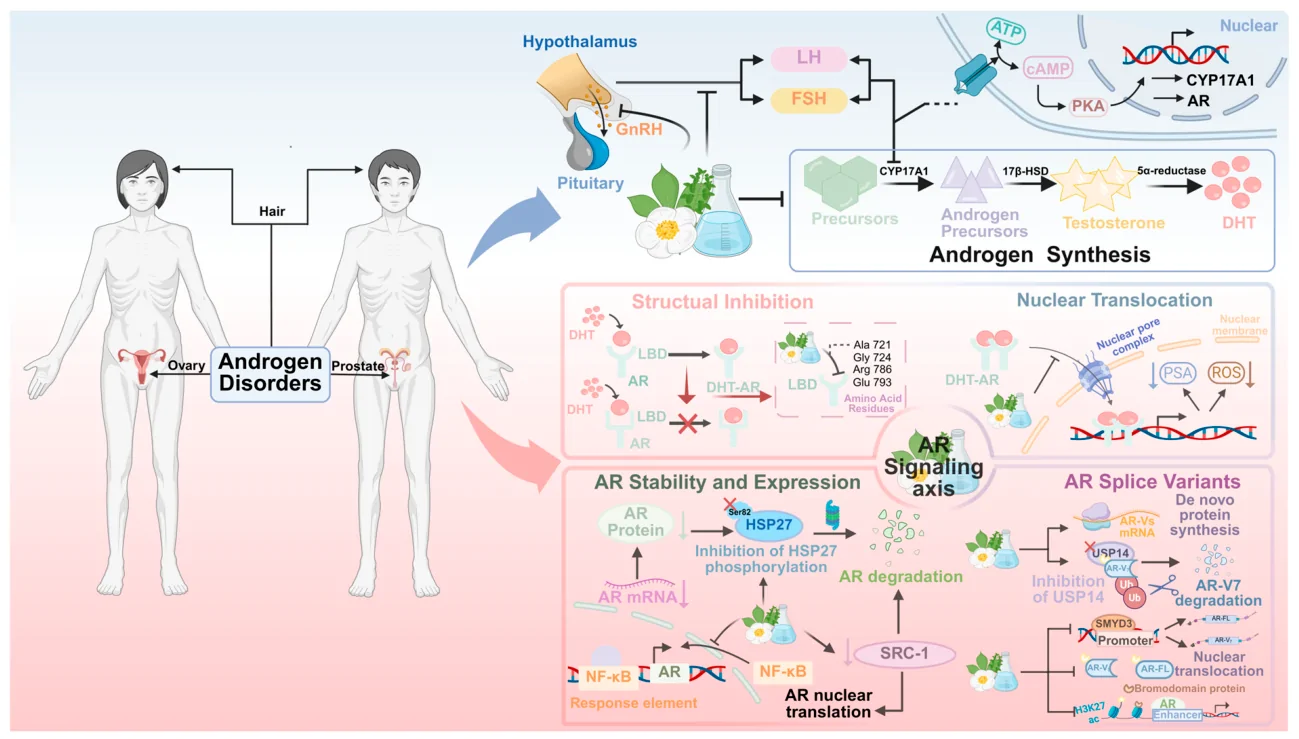
From androgen synthesis to AR signaling axis: dual regulation of plant extracts in androgenic disorders across multiple tissues. Plant extracts coordinately regulate androgen synthesis through modulation of the HPO axis and key steroidogenic enzymes, while inhibiting AR signaling axis via competitive structural inhibition, downregulation of AR expression and stability, blockade of nuclear translocation, and regulation of AR splice variant. Created with biorender.com.

AR is identified as a key member of the nuclear receptor superfamily [30]. Upon specific ligand binding, the AR translocates from the cytoplasm to the nucleus, where it binds to androgen response elements (AREs) to activate downstream signaling pathways [31]. Targeting this mechanism, anti-androgenic activities of plant extracts have been extensively demonstrated via competitively binding to AR, inhibiting the expression and nuclear translocation of AR and suppressing the transcription of downstream target genes (Figure 1).

#### 3.1.1. Structural Inhibition Targeting the Ligand-Binding Domain of AR by Plant Extracts

Structurally, AR primarily consists of four functional domains, with the C-terminal ligand-binding (LBD) domain being a primary therapeutic target [32]. *Serenoa repens* [24] crude ethanol extract has been demonstrated to exhibit anti-androgenic activity through potential structural interference with AR. Specifically, anthranilic acid ester derivatives of *Serenoa repens* fruit extract have been observed to directly bind to the LBD of the wtAR and suppress the T877A mutant without affecting the AR protein level. Furthermore, the stability of binding to the LBD may be influenced by the multi-active constituents of plant extracts. For instance, two isoflavones from soybean tempeh [33] crude ethyl acetate extract has been found to competitively inhibit the interactions between androgens and the AR by selectively binding to critical amino acid residues, specifically Ala721 and Gly724. Similarly, in the case of *Ipomoea batatas* (L.) Lamk [34] crude ethanol extract, bioactive constituents such as pyropheophorbide a, methyl pyropheophorbide a, hyperoside, and quercetin were identified via LC-MS/MS, all of which exhibited superior AR binding affinity in molecular dynamics simulations. Mechanistically, it was determined that hyperoside forms quadruple hydrogen bonds with the Arg786 and Glu793 residues, while methyl pyropheophorbide a binds stably via hydrophobic interactions, with both mechanisms significantly inhibiting DHT-induced AR activation.

#### 3.1.2. Regulation of AR Expression and Protein Stability by Plant Extracts

Another anti-androgenic strategy involves suppressing AR synthesis. The *Impatiens balsamina* L. crude ethyl acetate extract [35], the *Euphorbia ingens* ethyl acetate fraction [36] and the *Wedelia chinensis* [25] standardized ethanol extract have been shown to directly decrease AR mRNA levels. Unlike the former two extracts with active constituents that remain to be further characterized, the *Wedelia chinensis* extract was standardized through enrichment of wedelolactone, apigenin, and luteolin to approximately 70% by column chromatography, followed by HPLC-CAD- and LC-MS-based chemical characterization and quantification. Furthermore, as the nuclear factor-kappa B (NF-kB) response element is situated within the AR promoter region, the inhibition of NF-kB can suppress AR expression [37]. *Salvia miltiorrhiza* Bunge [38] standardized ethanol extract and *Cornus alba* [39] crude ethanol extract have been demonstrated to alleviate BPH by downregulating AR expression, associated with the inhibition of NF-kB transcriptional activity. Additionally, high expression of steroid receptor coactivator-1 (SRC-1) prevents AR degradation [40]. Downregulation of SRC-1 by *Abeliophyllum distichum* Nakai [41] crude ethanol extract correlates with reduced prostate indices and serum androgen levels. These results were also obtained with emphasis on *Astragalus membranaceus* [42] crude water extract and purple corn (*Zea mays* L.) crude ethanol extract [43] in testosterone propionate (TP)-induced BPH rat models. Except for targeting SRC-1, the *Astragalus membranaceus* extract reduces AR stability by inhibiting heat shock proteins 27 (HSP27) phosphorylation at the Ser82 amino acid residue, thereby disrupting the HSP27-AR interaction required for AR nuclear translocation and protection from proteasomal degradation. 

#### 3.1.3. Inhibition of AR Nuclear Translocation by Plant Extracts

Blocking AR nuclear translocation provides novel insights for targeting AR regulation. *Agaricus bisporus* crude water extract [44] has been shown in LNCaP and VCaP cells to suppress DHT-induced prostate-specific antigen (PSA) expression and cell proliferation by interrupting AR expression, nuclear-cytoplasmic distribution, and transactivation. Specifically, as the primary active constituent, a conjugated linoleic acid isomer has been demonstrated to be nearly twice as strong as the known AR antagonist. *Cucumis melo var. makuwa* [45] crude water extract has been shown to inhibit AR nuclear translocation, reduce AR protein expression and reactive oxygen species (ROS) production in DHT-induced human dermal papilla cells (HDPCs). The inhibition of AR translocation is primarily attributed to cucurbitacin A, the main active constituent in *Cucumis melo var. makuwa* leaf extract. Similarly, *Avicennia marina* [46] standardized ethanol extract and its active compound, avicequinone C, have been shown to significantly attenuate androgen-dependent AR activation in androgen-stimulated HDPCs by inhibiting the nuclear translocation of the DHT-AR complex. These results were directly observed through AR immunofluorescence staining and nuclear blue fluorescence assays.

#### 3.1.4. Downregulation of AR Splice Variants with Plant Extracts

Although clinical agents like enzalutamide effectively target the AR via the aforementioned mechanisms, their use is limited by inducing AR variants (AR-Vs) [47]. Nevertheless, a recent study in 22RV1 cells demonstrated that *Rubia cordifolia* [48] crude water extract overcomes drug resistance in castration-resistant prostate cancer (CRPC) by targeting both AR-Vs and the full-length androgen receptor (AR-FL). Mechanistically, *Rubia cordifolia* extract has been shown to target the de novo protein synthesis of AR-Vs, resulting in the specific downregulation of AR-Vs without altering mRNA expression levels. Concurrently, it has been observed that *Rubia cordifolia* extract indirectly inhibits the transcription of AR target genes by reducing bromodomain proteins expression and decreasing H3K27 acetylation. This epigenetic modulation significantly reduces AR binding to enhancers. In contrast, *Trametes robiniophila Murr*. [49] extract, a commercial formulation, has been shown to target AR-V7, a component of AR-Vs. Specifically, *Trametes robiniophila Murr.* extract promotes the proteasomal degradation of both AR-FL and AR-V7 by downregulating ubiquitin-specific protease 14. Furthermore, *Trametes robiniophila Murr*. extract downregulates AR-FL and AR-V7 mRNA levels via the SET and MYND domain-containing protein 3 (SMYD3) pathway, and subsequently directly inhibits the nuclear translocation and transcriptional activity of AR-FL and AR-V7. Intriguingly, *Rubia cordifolia* extract and *Trametes robiniophila Murr*. extract have shown synergistic effects when combined with classical anti-androgenic drugs. This suggests potential approaches for prostate cancer treatment. However, further chemical characterization is needed for *Trametes robiniophila Murr*. extract. RA-V was identified as a key compound of *Rubia cordifolia* extract, but its contribution to the whole extract activity remains unclear.

### 3.2. Inhibition of Androgen Synthesis via Plant-Mediated Regulation of Rate-Limiting Enzymes

In the studies of anti-androgenic activity of plant extracts, inhibition of AR and its signaling pathways is the classical strategy. Nevertheless, the sensitivity to low androgen concentrations is enhanced [50] by upregulating AR expression or inducing AR mutations. Consequently, targeting the androgen synthesis has emerged as another approach for regulating androgen signaling (Figure 1). Accumulating studies of anti-androgenic plant extracts have consistently indicated that the inhibition of androgen biosynthesis primarily targets three key rate-limiting enzymes [51,52,53], including 5α-reductase, cytochrome P450 17A1 (CYP17A1), and 17β-hydroxysteroid dehydrogenase (17β-HSD).

5α-Reductase catalyzes the rate-limiting conversion of testosterone into the potent androgen DHT. The inhibitory effects of plant extracts on this enzyme exhibit multi-pathway characteristics. Regarding direct inhibition, molecular docking analysis of BK002 [54], a formulation of *Achyranthes japonica* (Miq.) Nakai and *Melandrium firmum* Rohrb., predicted potential interactions and hydrogen bonds with the Tyr91, Met106, and Ser102 residue via the steroidal core structure. In cellular models, BK002 inhibited prostate cancer cell growth in PC3 and DU145 cells. However, these predicted binding modes require further biochemical validation to confirm direct enzyme inhibition. Similarly, *Avicennia marina* [46] extract demonstrated potent enzymatic inhibition in vitro. Its active constituent avicequinone C inhibited 5α-reductase activity by 67.71% at 10 μM. The extract effectively reduced DHT generation by competitively binding to the type-1 isoenzyme. Alternatively, plant extracts suppress the expression of steroid 5α-reductase (SRD5A) isoforms. For instance, *Oryza sativa* L. crude ethanol extract inhibited enzymatic activity by 60% in rat liver microsomes while significantly downregulating *SRD5A2* and *SRD5A3* expression. Meanwhile, *Cucumis sativus* crude water extract [55], *Celtis choseniana* Nakai crude methanol extract [56], and *Musa* flowers crude water extract [57] have been shown to effectively reduce DHT production and alleviate pathological damage by specifically targeting *SRD5A1* or *SRD5A2*. Crucially, phytotherapeutic inhibition often exhibits isoform specificity. For example, *Rauwolfia vomitoria* [58] extract specifically targets the 5α-reductase type-1, whereas *Glycyrrhiza uralensis* Fischer crude water extract [26], *Paeonia suffruticosa* pollen crude ethanol extract [59], and *Astragalus membranaceus* [60] preferentially inhibit the type-2 isoenzyme. Given the distinct tissue distribution of these isoenzymes, such selectivity provides a theoretical foundation for precision therapeutic interventions across different disease models.

Upstream of 5α-reductase, androgen biosynthesis relies on the sequential catalysis of CYP17A1 and 17β-HSD. Targeting the dual hydroxylase and lyase activities of CYP17A1 is a core strategy for blocking precursor generation. For instance, epigallocatechin-3-gallate from *Camellia sinensis* L. [61] whole dietary material, was predicted to interact with CYP17A1 through hydrogen bonding and Van der Waals interactions. However, whether these interactions affect the hydroxylase and lyase activities of CYP17A1 remains to be determined. Except for potential direct enzymatic targeting, *Terminalia chebula* [62] crude ethanol extract exerts anti-androgenic effects by downregulating CYP17A1 mRNA and protein expression in PCOS models. Regarding 17β-HSD, the balance between activating and inactivating isoenzymes contributes to androgen homeostasis [63]. Crude extracts from *Annona muricata* fruit [64] and soybean tempeh have demonstrated efficacy in suppressing 17β-HSD activity. Specifically, soybean isoflavones [33,65] selectively bind to critical residues of 17β-HSD and inhibit the pro-activation isoenzymes in PCOS rats. This results in a dose-dependent reduction in serum testosterone levels and the restoration of normal estrous cycles.

Except for direct enzymatic regulation, androgen biosynthesis is also regulated by the hypothalamic–pituitary–ovarian (HPO) axis. Aberrant LH pulsatility hyperactivates the thecal luteinizing hormone receptor (LHR)-cyclic AMP (cAMP)-protein kinase A (PKA) signaling pathway, driving the overexpression of CYP17A1 and 17β-HSD [66]. Unlike single-target agents that may trigger compensatory feedback, plant extracts modulate the HPO axis through multiple pathways, as demonstrated in letrozole- and DHEA-induced PCOS rat models. For instance, phenolic constituents from *Inonotus obliquus* [67] commercial water extract ameliorate the abnormal excitation of hypothalamic GnRH neurons through antioxidant and anti-inflammatory effects. *Calendula officinalis* [68] crude hydroalcohol extract directly regulates the pulsatile rhythm of pituitary luteinizing hormone (LH) secretion. Flavonoids from *Garcinia cambogia* [27,69] commercial water extract improve insulin resistance to attenuate the overstimulation of the ovaries. These findings suggest that different plant extracts converge on restoring androgen homeostasis through complementary regulation of hypothalamic, pituitary, and ovarian functions, despite acting through distinct upstream mechanisms. This multi-target advantage not only enriches the regulatory network of the anti-androgenic activity of plant extracts but also provides potential directions for PCOS.

### 3.3. Amplification of Anti-Androgenic Efficacy via Plant-Mediated Signaling Pathway Crosstalk

**Figure 2:**
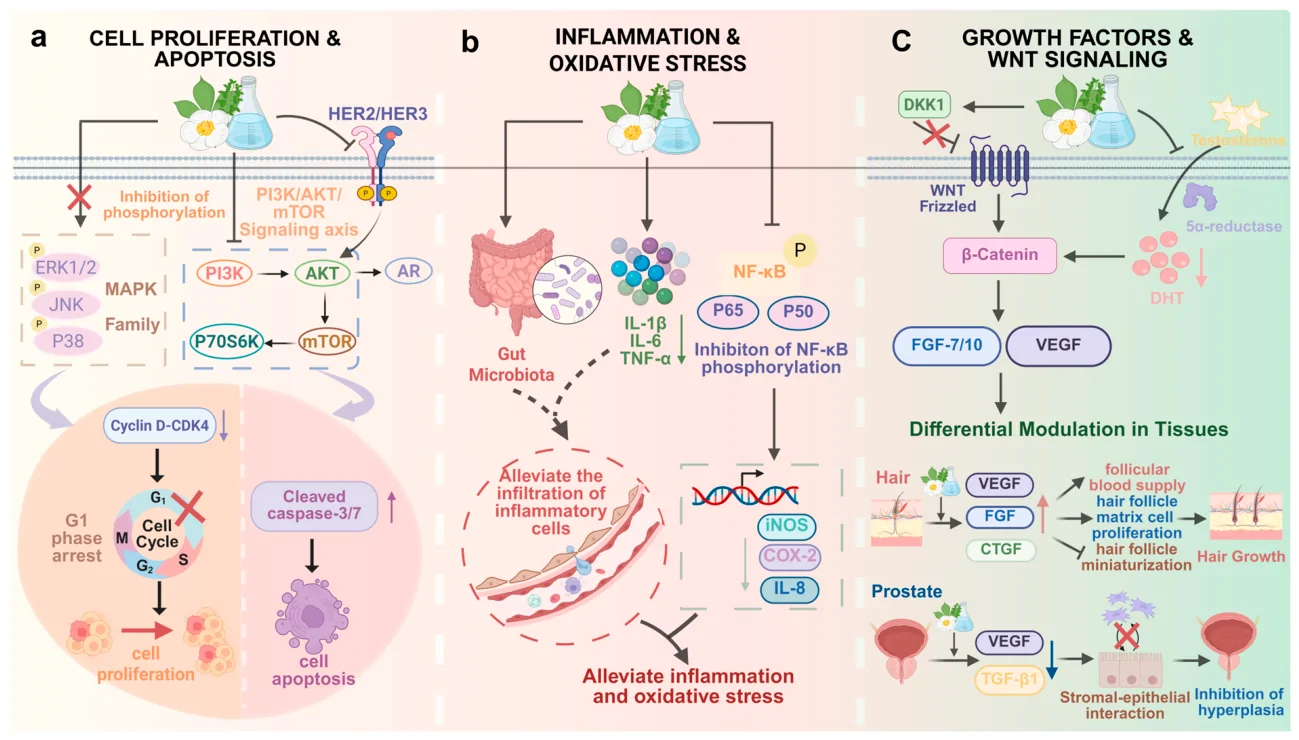
Multidimensional anti-androgenic networks of plant extracts: integrating cell proliferation and apoptosis, inflammatory microenvironments, and tissue-specific modulation of growth factors. (**a**) Plant extracts regulate androgen-related cell growth through modulation of HER2/HER3, PI3K/Akt/mTOR, and MAPK signaling, resulting in cell cycle arrest and apoptosis. (**b**) Plant extracts attenuate androgen-related inflammatory microenvironments and oxidative stress via inflammatory factors and NF-κB signaling pathway, together with the regulation of gut microbiota. (**c**) Plant extracts differentially regulate growth factor and Wnt/β-catenin signaling in tissue-specific contexts. Created with biorender.com.

In addition to modulating androgen biosynthesis and the AR signaling axis, plant extracts amplify their anti-androgenic efficacy through crosstalk with extensive signaling networks. These coordinated modulations primarily converge on inflammation, oxidative stress, and growth factor signaling, ultimately regulating cell proliferation via apoptosis induction and cell cycle arrest in androgen-sensitive tissues. These interconnected regulatory networks are schematically summarized in Figure 2.

#### 3.3.1. Modulation of Cell Proliferation and Apoptosis by Plant Extracts

Plant extracts modulate cell growth primarily through the mammalian target of rapamycin (mTOR)/phosphatidylinositol 3-kinase (PI3K)/Akt (also known as protein kinase B) and mitogen-activated protein kinase (MAPK) signaling pathways. The mTOR complex, a downstream effector of PI3K/Akt, is a critical target for inhibiting androgen-dependent cell growth [70]. For instance, *Poncirus trifoliata* (L.) Raf. crude methanol extract [71] significantly downregulates the mTOR/P70S6K axis in LNCaP and PC3 cell lines, without affecting pAkt levels. This signaling alteration was associated with G0/G1 cell cycle arrest in LNCaP cells. Treatment with 12.5–25 μg/mL extract for 24 h increased the G1-phase population from 68.93% in controls to 84.14–89.14%. In contrast, *Wedelia chinensis* [25] extract disrupts the human epidermal growth factor receptor 2/3 (HER2/3) and Akt signaling, enhancing the anti-tumor response to androgen deprivation (ADT) in prostate cancer models. Specifically, in CRPC cell models, androgen deprivation stabilizes AR signaling by inducing a significant upregulation of HER3 protein. However, *Wedelia chinensis* extract offers a novel therapeutic strategy to overcome the insensitivity of CRPC to both ADT and the antiandrogen agent enzalutamide by suppressing AR and HER2/3 signaling. The MAPK signaling cascade represents another group of serine/threonine kinases, including the extracellular signal-regulated kinases (ERK), c-Jun N-terminal kinase (JNK), and p38 mitogen-activated protein kinase (p38) [72]. Numerous studies have demonstrated that various plant extracts modulate MAPK/ERK pathways to inhibit cell growth. *Cornus alba* [39] and *Houttuynia cordata* Thunb. crude ethanol extracts [73] have been shown to effectively reduce the phosphorylation of ERK1/2, JNK, and p38 proteins within the MAPK family. Intriguingly, treatment with 400 μg/mL Cornus alba crude ethanol extract inhibited WPMY-1 cell proliferation by approximately 50% through downregulation of the cyclin D-dependent kinase 4/Cyclin D1 complex, leading to G1-phase cell cycle arrest. Conversely, *Houttuynia cordata* Thunb. extract primarily induces apoptosis by upregulating the expression of cleaved caspase-3 and cleaved caspase-7 in a dose-dependent manner.

#### 3.3.2. Attenuation of Inflammatory Responses and Oxidative Stress by Plant Extracts

The NF-κB transcription factor family is pivotal in linking inflammation with androgenic pathology [74]. The combination of *Curcumae Radix* and *Syzygium aromaticum* [75] crude extracts have been shown to regulate inflammatory responses via NF-κB in TP-induced BPH rat model. Specifically, these extracts indirectly block the transcription of downstream pro-inflammatory genes, including *iNOS*, *COX-2*, and *IL-8*, and mitigate oxidative and inflammatory stress in prostate tissue, within inhibition of NF-κB phosphorylation. Notably, this combination exhibits potency at low doses.

Additionally, plant extracts target key inflammatory mediators that associated with NF-κB, such as tumor necrosis factor-α (TNF-α). In a computational virtual screening and molecular docking study, 4,5,8-tetramethyl-2,3-dihydrochromen-2-ol from *Nothapodytes nimmoniana* (J. Graham) Mabb. [76] crude methanol extract exhibited significant binding affinity for both TNF-α and human estrogen receptor-alpha. Its binding stability was found to be superior to that of clomiphene, a selective estrogen receptor modulator. The inhibitory effects of plant extracts on TNF-α have also been validated in both in vivo and in vitro. For instance, in a TP-induced BPH rat model, *Paeonia suffruticosa* pollen [59] extract significantly reduces the expression levels of TNF-α, IL-6, and IL-1β in serum and prostate tissue, alleviates the infiltration of inflammatory cells, and synergistically modulates gut microbiota to indirectly mitigate systemic and local inflammation [77]. Similarly, in AGA models, crude extracts from *Musa* flowers [57], *Angelica sinensis* and *Platycladus orientalis* [78] have been shown to attenuate inflammatory responses, accompanied by reduced scalp inflammation or erythema. These effects are associated with the upregulation of hair growth factors, leading to increased hair thickness and follicle count and a higher proportion of anagen-phase follicles. Despite their different chemical profiles, a common pattern appears to be the concurrent attenuation of the inflammatory microenvironment and activation of regenerative signaling. This pattern is also supported by *Justicia procumbens* [79], in which 4-CQA was identified as a bioactive constituent that promotes β-catenin signaling and the expression of FGF7, FGF10, KGF, and VEGF.

#### 3.3.3. Differential Modulation of Growth Factors and Wnt Signaling by Plant Extracts

Growth factors act as critical mediators in the distinct microenvironments of androgen-related disorders [80]. Interestingly, plant extracts exhibit differential regulation of these factors depending on the pathology. In AGA, upregulation of growth factors is therapeutic. For example, *Lepidium sativum* L. [81] crude water extract significantly upregulates the expression levels of vascular endothelial growth factor (VEGF), CTGF, and fibroblast growth factor (FGF) in skin tissues, which improves follicular blood supply, promotes hair follicle matrix cell proliferation, and reverses DHT-induced hair follicle miniaturization. Concurrently, molecular docking analysis predicted that flavonoids from *Lepidium sativum* L. extract may interact with the androgen receptor. These predicted interactions, together with the observed regulation of androgen-related responses, may contribute to reduced DHT-associated follicular changes and improved hair growth. Conversely, in BPH, downregulation of growth factors is required to inhibit hyperplasia. While exhibiting 5α-reductase inhibitory activity, *Panicum dichotomiflorum* [82] crude extract significantly downregulates TGF-β1 and VEGF mRNA in BPH rat models. This prevents the stromal–epithelial interactions that exacerbate androgen-mediated proliferation. These differential results suggest that plant extracts modulate upstream AR signaling via tissue-specific co-regulator recruitment in stromal cells. This selective regulation alters local growth factor synthesis, driving context-dependent responses in adjacent epithelial cells.

The Wnt signaling pathway plays a crucial role in promoting hair growth [83]. *Mangifera indica* [84] crude water extract has been demonstrated to activate the Wnt signaling pathway via downregulating SRD5A2 and DKK1 in a depilated mouse model. Similarly, the aqueous extract of *Justicia procumbens* prolongs [79] the hair follicle anagen phase by activating the Wnt/β-catenin signaling pathway to regulate the expression of FGF-7/10 and VEGF.

In summary, the biological effects of plant extracts are associated with their abundance of bioactive constituents [85], including flavonoids [86], phenolic acids, and terpenoids. These compounds precisely target AR signaling and androgen biosynthesis. Concurrently, they may influence inflammatory and oxidative stress-related pathways, contributing to a broader regulatory network. Notably, identical growth factors may exhibit differential regulatory roles across distinct androgen-related disorders. Leveraging their capacity for multi-pathway modulation, plant extracts adaptively address the functional requirements of growth factors within specific disease microenvironments, thereby eliciting divergent outcomes on the same molecular target. This insight may provide potential perspectives for the future application of plant extracts in targeting diverse pathological conditions.

## 4. Biological and Translational Perspectives of Plant Extracts Targeting Androgen-Related Disorders

Androgens are classified into five distinct forms based on their relative potency. Predominantly synthesized in the adrenal glands and gonads [87], these molecules undergo metabolic conversion and processing in various peripheral tissues, including the prostate, skin, and adipose tissue [88,89]. Consequently, the dysregulation or aberrant activation of androgen signaling frequently serves as a primary pathogenic factor underlying diverse disorders. Excessive levels of DHT drive the aberrant cellular proliferation associated with BPH and PCa [90], or induce follicular miniaturization in AGA [91], whereas hyperandrogenemia in females is a key precipitating factor for PCOS [92]. Compared with some synthetic anti-androgenic agents, plant extracts have attracted interest due to their diverse bioactive constituents and potential advantages observed in preclinical studies. Leveraging these properties, plant extracts exert anti-androgenic activity across these four pathologies by modulating relevant signaling pathway crosstalk. In recent years, an increasing number of plant extracts have been investigated for their toxicity profiles, mechanisms of action, research methodologies, and clinical potential in the targeted intervention of androgen disorder-related diseases.

### 4.1. Management of PCa Progression and Drug Resistance with Plant Extracts

The progression of PCa is intrinsically linked to androgens and the AR signaling pathway, with the majority of early-stage tumors exhibiting androgen-dependent proliferation. As a critical downstream target of the AR, PSA levels are widely recognized as a pivotal biomarker for the PCa diagnosis and prognosis [93]. As summarized in Table 1, plant extracts have been evaluated for anti-androgenic activity targeting proliferation, migration and invasive phenotypes of prostate epithelial cells. Among these, *Algerian propolis crude extracts* [94], *Aspalathus linearis* [95], *Hippophae rhamnoides* L. fractionated extracts [96] and *Eriobotrya japonica* crude extracts [97] have been demonstrated to attenuate PSA protein expression in LNCaP cells, an effect frequently accompanied by the concomitant inhibition of AR activity.

While conventional clinical modalities for PCa management are well-established, the transition to CRPC represents a primary therapeutic challenge. Clinically, CRPC is characterized by initial androgen-dependent tumor proliferation [98]. Although androgen ADT effectively reduces serum testosterone to castrate levels, CRPC is characterized by persistent tumor progression despite the blockade of androgen synthesis [99]. In castration-resistant models, *Eriobotrya japonica* extract demonstrates therapeutic potential by targeting the AR axis and inducing apoptosis. Beyond conventional anti-androgenic pathways, *Eriobotrya japonica* extract also targets aberrant de novo fatty acid and lipid biosynthesis. The extract inhibited LNCaP and C4-2 cell growth with IC_50_ values of 42.90 and 46.28 μg/mL, respectively, after 48 h treatment. This growth inhibition was accompanied by reduced SREBP-1 and fatty acid synthase (FASN) expression, resulting in decreased intracellular fatty acid levels and lipid accumulation required for tumor maintenance.

Furthermore, enzalutamide, as a second-generation AR antagonist, serves as a first-line targeted therapeutic agent for CRPC. However, with prolonged administration, enzalutamide resistance has emerged as a predominant clinical challenge in the management of CRPC [100]. Pharmaceutical-grade enriched Aspalathus linearis [95] extract has been demonstrated to significantly inhibit the survival and proliferation of enzalutamide-resistant PCa cells, with an IC_50_ value of 105.3 μg/mL. Its potent inhibitory activity is primarily mediated by the suppression of c-Myc and the modulation of AR stability. Mechanistically, treatment with Aspalathus linearis extract significantly downregulates c-Myc protein expression in enzalutamide-resistant prostate cancer cells.

**Table 1:** Anti-androgenic activity of plant extracts targeting proliferation, migration and invasive phenotypes of prostate epithelial cells.

Extract	Plant Part	Extract Type	Chemical Characterization	Research Methods		Key Findings	Ref.
				Model	Dosage Regimen		
*Aspalathus linearis*	Leaf	Pharmaceutical-grade enriched extract	HPLC-characterized flavonoids	C4-2 MDV3100r, HUVEC and HEK293 cells	0–100 μg/mL; 0–200 μg/mL	Inhibited AR and its signaling Inhibited cell cycle regulatory proteins Inhibited the PI3K-Akt signaling Inhibition of c-Myc	[95]
*Wedelia chinensis*	Whole plant	Standardized ethanol extract	HPLC-CAD and LC-MS quantification of wedelolactone, apigenin and luteolin	22Rv1 cells xenograft tumor mice model	4 or 40 mg/kg	Downregulated AR expression and inhibited the AR signaling pathway Induced caspase-dependent apoptosis Inhibited the HER2/3-Akt pathway	[25,101]
				LNCaP, 22Rv1 and PC-3 cells	10–50 μg/mL		
*Eriobotrya japonica*	Leaf	Crude ethanol extract	HPLC-characterized triterpenes, tormentic acid	C4-2 cells xenograft tumor mouse model	20 or 40 mg/mL, 45 days	Attenuated PCa cell growth, migration, and invasion Regulated lipid metabolism in PCa cells Blocked the SREBP-1 and AR axis Activated the apoptotic pathway	[97]
				C4-2, LNCaP cells	20–80 μg/mL		
*Angelica gigas*	Root	Crude ethanol extract	HPLC-characterized decursin	LNCaP cells	20–100 μg/mL	Inhibited cell proliferation Inhibited the PSA expression Inhibited AR nucleus translocation Downregulates AR protein abundance	[102]
*Euphorbia ingens*	Root	Ethyl acetate fraction	GC-MS-characterized terpenoid-rich phytochemical profile	DU-145 and Vero E6 cells	0–200 μg/mL	Inhibited DU-145 proliferation Induction of apoptosis Suppression of cell cycle Regulated AR and PI3K-Akt/MAPK/p53 pathways	[36]
*Poncirus trifoliata* (L.) Raf. Seed	Seed	Crude methanol extract	HPLC-DAD-characterized flavanone-rich profile	LNCaP and PC3 cells	1–100 μg/mL	Inhibited LNCaP cell viability Inducing G0/G1 phase arrest Decreased the AR Regulated the MAPK and AKT/mTOR pathway	[71]
*Oryza sativa* L.	Caryopsis	Crude ethanol extract	Total phenolics and HPLC-characterized anthocyanins	Transgenic rat for the adenocarcinoma of the prostate model	0.2% or 1% PRE-HIF supplied diet, 10 weeks	Inhibition of cell proliferation Induction of G1 cell cycle arrest Inhibits Cell Growth Pathways Inhibited 5α-R activity Regulated AR production and AR-activated pathway	[103]
				LNCaP and PCai1 cells	75–400 μg/mL		
*Astragalus membranaceus*	Root	Crude water extract	HPLC-characterized isoflavonoids	22Rv1 cells xenograft tumor mice model	37.1 mg/kg, gavage, 4 weeks	Inhibited LNCaP viability Blocking AR nuclear translocation Inhibition of HSP27 phosphorylation ROS-mediated apoptosis Activated caspase-3/PARP pathway	[42]
				LNCaP, PC3, DU145, and RWPE1 cells	0–800 μg/mL		
*Rubia cordifolia*	Not specified	Crude water extract	LC-MS and NMR identification of RA-V as the key active compound	22RV1 cells xenograft tumor mouse model	500 mg/kg, oral, 7 days	Inhibited xenograft growth with enzalutamide Dual inhibited AR/GR activity Downregulated AR and AR-V proteins Epigenetic regulation	[48]
				22RV1, LNCaP and PC3 cells	30–600 μg/mL		
*Impatiens balsamina* L.	Seed	Crude ethyl acetate extract	UHPLC-MS/MS chemical profiling (218 compounds identified)	PC-3 cells xenograft tumor mouse model	0–0.24 g/mL, 100 μL/mouse, gavage, 21 days	Reduced xenograft weight Inhibited AR^+^/AR^−^ PCa cell proliferation Inducing G0/G1-phase arrest Inhibited AR expression and transcription Upregulation of ATF3 in the nucleus	[35]
				LNCaP, 22Rv-1, PC-3 and DU145 cells	0–0.2 mg/mL		
*Agaricus bisporus*	Fruiting body	Crude water extract	GC-identified CLA-9Z11E	PDX tumor xenograft tumor mice model	200 mg/kg, by gavage daily	Inhibited cell proliferation Suppressed PSA expression Inhibited AR transcription, transactivation and nuclear translocation Reduced the mRNA and protein levels of AR target genes	[44]
				LNCaP, VCaP, PC-3, and RWPE-1 cells	1–20 μL/mL		
*Houttuynia cordata* Thunb.	Whole plant	Crude ethanol extract	HPLC profiling and quantification of phenolic and flavonoid compounds	Transgenic prostate rat and CRPC xenograft mice model	0.2% or 1% HCT mixed diet, 10 weeks	Suppress EMT and PCa Cell migration through STAT3/Snail/Twist Pathways Activated caspase-mediated apoptosis Inhibited Akt/ERK/MAPK pathways	[73]
				LNCaP and PCai1 cells	0–200 μg/mL		
*Achyranthes japonica* Nakai; *Melandrium firmum* Rohrb.	Not specified	Crude herbal combination extract	Reported ecdysterone, inokosterone and 20-hydroxyecdysone	Molecular docking and in silico analyses		Predicted interaction with 5α-R and CYP17A1 Relieved the epigenetic silencing of tumor suppressor genes Reduced tumor immune escape Activated the apoptotic pathway	[54]
*Cucumis sativus*	Seed	Crude water extract	Qualitative phytochemical screening detected flavonoids, tannins and saponins	Testosterone-induced BPH rat model	125–1000 mg/kg, gavage, 4 weeks	Reduce the prostate volume Inhibited DU145/PC3 proliferation Inhibited the 5α-reductase Anti-inflammatory activity	[55]
				LNCaP, PC-3 and DU145 cells	2.5–100 μg/mL		
*Camellia sinensis* L.	Leaf	Dietary material	LC-MS metabolomics dataset containing catechins	Molecular docking; Pharmacokinetic and toxicity evaluations; Molecular dynamics simulations		Docking- and MD-predicted interaction with CYP17A1	[61]
*Trametes robiniophila* Murr.	Fruiting body	Commercial formulation	Reported protein-bound polysaccharides	22Rv1 cells xenograft tumor mice model	50 mg in 100 μL normal saline, oral, 25 days	Induced PCa cells apoptosis and G2/M arrest Regulated AR-FL and AR-V7 degradation, nuclear translocation and transcriptional activity on their target genes	[49]
				LNCaP and 22Rv1 cells	0–10 mg/mL		
*Hippophae rhamnoides* L.	Leaf	24 solvent systems of polarities fractionated extract	Not characterized	LNCaP and C4-2 cells	3.12–50 μg/mL	Inhibited LNCaP and C4-2 cells proliferation Inhibited cancer cell migration Inhibition of AR nuclear translocation	[96]

Notably, while c-Myc overexpression exacerbates the resistant phenotype in C4-2 MDV3100r cells, c-Myc knockdown in PC-3 cells significantly enhances their sensitivity to the extract. These findings offer novel therapeutic perspectives for addressing the clinical challenge of enzalutamide resistance in PCa patients.

### 4.2. Amelioration of BPH Pathology and Clinical Evidence Validation with Plant Extracts

BPH represents a prevalent androgen-dependent pathology affecting aging men except for PCa. Both in vivo and in vitro studies have demonstrated that various plant extracts alleviate the pathological manifestations of BPH by targeting androgenic pathways, inflammatory cascades, and apoptotic mechanisms (Table 2). *Taraxacum officinale* crude ethanol extract [104], *Glycyrrhiza uralensis* Fischer crude water extract [26], *Juglans regia* crude ethanol extract [105], *Panax ginseng* crude oil extract [106], and *Citrus reticulata* crude ethanol extract [107] have been demonstrated to significantly reduce testosterone-induced absolute and relative prostate weights. Among these, *Taraxacum officinale* extract at a dosage of 300 mg/kg has been observed to attenuate prostate weight gain by 22.6%. This effect showed a similar trend to that observed in the finasteride-treated group. However, specific active constituents of this extract remain to be further characterized. Histological analyses further demonstrated that these extracts reduced epithelial thickness, attenuated papillary hyperplasia, and restored prostate luminal structure. Interestingly, different studies applied distinct histological evaluation approaches to assess prostate improvement. Specifically, studies on *Taraxacum officinale* extract used a comprehensive multiparametric scoring system [108]. This system evaluated luminal and acinar morphology, interacinar spacing, stromal changes, epithelial characteristics, and nuclear morphology [109]. In contrast, *Citrus reticulata* fruit peel extract was evaluated using the modified Gleason grading system established by the International Society of Urological Pathology, which is commonly applied for assessing prostate cancer differentiation [110]. Under intervention with *Citrus reticulata* fruit peel extract, the primary and secondary pathological scores of prostate tissues in BPH rats were significantly lower than those of the model group. This result highlights the therapeutic potential in managing BPH [107].

Although clinical histopathological scoring has been introduced in research targeting BPH with plant extracts, data regarding their clinical application remain insufficient. A double-blind trial of *Serenoa repens* [111] was conducted to bridge this gap in clinical evidence. In this trial, 94 BPH outpatients were assigned to receive placebo or 320 mg/day of PA109. PA109 is a tablet formulation containing 80 mg of liposterolic *Serenoa repens* extract. After a 30-day treatment course, patients were evaluated based on indicators including nocturia, dysuria severity, urine flow rate, post-void residual volume, patient self-assessment, and physician evaluation. The results indicated that PA109 improved BPH-related symptoms and urinary function. Moreover, PA109 was well tolerated, with fewer reported side effects than placebo.

**Table 2:** Anti-androgenic activity of plant extracts targeting benign prostate enlargement and stromal–epithelial crosstalk.

Extract	Plant Part	Extract Type	Chemical Characterization	Research Methods		Key Findings	Ref.
				Model	Dosages Regimen		
*Salvia miltiorrhiza* Bunge	Root	Standardized ethanol extract	HPLC-quantified cryptotanshinone, tanshinone I/IIA, dihydrotanshinone	(TP)-induced BPH rat model	20–80 mg/kg, oral, 4 weeks	Dose-dependently improves BPH Induced apoptosis by regulating the cell cycle Decreased excessive free radical production and inflammatory factor activation Inhibited the NF-κB and AR signaling pathway Activated the Nrf-2/HO-1 pathway	[38]
				LNCaP and BPH-1 cells	5–20 μg/mL		
*Glycyrrhiza uralensis* Fischer	Not specified	Crude water extract	LC/MS-identified glycyrrhizin	(TP)-induced BPH rat model RWPE-1 cells	80 or 800 mg/kg, oral, 4 weeks; 1–1000 μg/mL	Reduced the enlarged prostate weight, prostate index, prostate epithelial thickness Decreased the serum level of DHT Downregulated the AR and 5AR2 Induced apoptosis Protected against Fi-induced sperm loss	[26]
*Juglans regia*	Fruit	Crude ethanol extract	HPLC-ESI-Q-TOF-MS identified phenolic acids, flavonoids and galloyl	(TE)-induced BPH rat model	50 or 100 mg/kg, oral, 4 weeks	Reversed increase in body weight and food intake Antiproliferative activity Decreased the levels of testosterone, PSA Antioxidant Anti-inflammatory	[105]
				DU145, PNT1A cells	62.5–2000 μg/mL		
*Paeonia suffruticosa*	Pollen	Crude 70% ethanol extract	UPLC-QTOF/MS and UPLC-TQ/MS quantification of terpenoids, flavonoids and phenolic acids	(TP)-induced BPH rat model	625 mg/kg, gavage, 4 weeks	Decreased prostate weight and epithelial hyperplasia Reduced the levels of testosterone, DHT, 5α-R, and PSA Reduced oxidative damage Inhibition of inflammatory factors Regulated the gut microbiota disorder Regulated SCFAs metabolism	[59]
*Curcumae Radix* and *Syzygium aromaticum*	Root	Crude water extract	HPLC-characterized for curcuminoids	(TP)-induced BPH rat model	50–200 mg/kg, oral, 6 weeks	Inhibited the increase in prostate weight and thickness of prostate epithelium Inhibition of cell proliferation Decreased the level of PSA and DHT Reduced inflammatory cytokine Suppressed the expression of PSA and AR Reduced reactive oxygen species and NOX4-iNOS-COX2 axis	[75]
				RWPE-1 cells	10–100 μg/mL		
*Rauwolfia vomitoria*	Root bark	HPLC quality-controlled alkaloid-rich ethanol extract	HPLC quality-controlled β-carboline and indole alkaloids	(TP)-induced BPH rat model	20 mg/kg, oral, 28 days	Inhibited the prostate epithelial cells proliferation Reduced the proliferation markers Inhibited AR signaling pathway	[58]
*Panicum dichotomiflorum*	Leaf and stem	Crude extract	UPLC-UV-QTOF/M identification of flavonoid glycoside and steroidal saponin	Testosterone-induced BPH rat model	150 mg/kg, gavage, 4 weeks	Reduced prostate weight and the thickness of glandular epithelium Alleviated prostate cell growth by apoptosis Decreased Androgen and 5α-R Reduced growth factors and inflammatory cytokines	[82]
*Zea mays* L.	Husk and cob	Crude 30% ethanol extract	HPLC-characterized anthocyanins	(TP)-induced BPH rat model	2–50 mg/kg, oral, 4 weeks	Alleviated prostate weight and epithelial thickness Modulated cell proliferation and apoptosis Reduced androgen levels in the prostate Inhibition of AR signaling pathway Inhibited the PI3K/Akt pathway	[43]
				WPMY-1 cells	0–1000 μg/mL		
*Panax ginseng*	not specified	Crude supercritical CO_2_ oil extract	GC-FID characterization of fatty acids, ginsenosides and phytosterols	TP-induced BPH rat model	25–200 mg/kg, 8 weeks	Decreased weight and epithelial cell thickness Inhibition of proliferation Reduced the levels of DHT, 5α-R and PSA	[106]
				BPH-1 cells	0–500 μg/mL		
*Psoralea corylifolia* L.	Seed	Crude 30% ethanol extract	HPLC-characterized psoralen and psoralidin	(TP)-induced and castrated BPH rat model	50 or 100 mg/kg, oral, for 4 weeks	Decreased prostate weight and epithelium thickness Induced AR-mediated cell apoptosis Reduced the expression of AR androgen signaling-related marker	[112]
				WPMY-1 and RWPE-1 cells	62.5–500 μg/mL		
*Citrus reticulata*	Fruit peel	Crude ethanol extract	Qualitative phytochemical screening detected alkaloids, tannins, flavonoids, terpenoids and saponins	Testosterone-induced BPH rat model	50–200 mg/kg, oral, for 4 weeks	Reduced prostate weight Attenuated atresia of the prostatic glands, stromal fibrosis, and mast cell infiltration Increased glandular secretion Reduced the levels of testosterone, CRP, PSA, and white blood count Attenuated inflammatory activity	[107]
*Serenoa repens*	Fruit	Crude ethanol extract	Reported methyl ester and methyl anthranilate	LNCaP cells	MA 300 μM; E1-E4 30 μM	Reduced DHT production Inhibited testosterone 5α-R activity Required the AR-LBD to exhibit an anti-androgenic effect Inhibited the transactivation of both the wt AR and the AR-T877A mutant	[24,111]
				CV1 cells	300 μg/μL		
*Abeliophyllum* *distichum* Nakai	Leaf	Crude 70% ethanol extract	Reported flavonoids and polyphenols	(TP)-induced BPH rat model	100 mg/kg, oral, 4 weeks	Reduced the prostate size and prostate epithelial cell thickness Inhibited prostate cell proliferation Induced cell apoptosis. Suppressed the levels of DHT and 5α-R Reduced the expression of growth factors Inhibited AR signaling pathway Inactivated the PI3K/Akt pathway	[41]
				LNCaP cells	100 μg/mL		
*Taraxacum officinale*	Whole plant	Crude 70% ethanol extract	Reported sesquiterpenoid lactones, phenolic acids and flavonoids	(TP)-induced BPH rat model	100 or 300 mg/kg, oral, 4 weeks	Lowered serum levels of testosterone and DHT Modulated androgen signaling Induced apoptosis	[104]
*Cornus alba*	Not specified	Crude ethanol extract	Reported cornusiin A and camptothin B	(TP)-induced BPH rat model	50 or 100 mg/kg	Induced G1-phase cell cycle arrest Modulated 5 AR/AR axis-mediated signaling Regulated Akt and MAPK signaling pathways Reduce the binding ability of the NF-κB motif	[39]
				WPMY-1, RWPE-1 cells	0–400 μg/mL		
*Asparagi radix*	Root	Crude 70% ethanol extract	Remains to be elucidated	(TP)-induced BPH rat model	200 or 400 mg/kg, oral, 7 weeks	Reduced the prostate weight, lumen size, and epithelial thickness Reduced levels of testosterone, DHT, and PSA Inhibition of 5α-reductase activity Inhibited AR transactivation and its signaling pathway	[60]
*Annona muricata*	Fruit pulp	Freeze-dried crude fruit juice extract	Remains to be elucidated	(TP)-induced BPH rat model	1600–2400 mg/kg, 21 days	Reduced the level of testicular glycogen, testicular cholesterol, and testicular zinc Inhibited 3β-HSD and 17β-HSD activity Decreased the expression of DHT and PSA	[64,113]

### 4.3. Stimulation of Hair Regrowth in AGA with Plant Extracts: Insights from Research Models

Increased local DHT and enhanced follicular sensitivity are major causes of AGA. This condition is phenotypically characterized by progressive follicular miniaturization. The dermal papilla (DP), representing the sole dermal component of the hair follicle and composed of fibroblasts ensheathed by the follicular epithelium, serves as the central regulator of the hair cycle and a direct determinant of hair shaft diameter [114]. Consequently, the loss or dysfunction of the DP precludes sustained hair growth. Given this pivotal role, HDPCs have established themselves as the premier in vitro model for evaluating the therapeutic potential of plant extracts in AGA intervention. Table 3 summarizes extracts promoting hair growth target follicle miniaturization. *Allium macrostemon* Bunge crude water extract [115] and *Stauntonia hexaphylla* crude extract [116] have been corroborated to enhance the proliferation of HDPCs. Specifically, *Allium macrostemon* Bunge extract exhibited maximal proliferative efficacy at a concentration of 30 μg/mL. This effect is attributed to inhibiting HDPCs apoptosis while synergistically activating the Wnt/β-catenin signaling pathway. This mechanism is further substantiated by findings where the proliferative effect of *Allium macrostemon* Bunge extract on HDPCs was abrogated following treatment with a specific inhibitor of β-catenin. Conversely, *Stauntonia hexaphylla* extract primarily modulates the HDPCs cycle. Specifically, *Stauntonia hexaphylla* extract has been observed to facilitate the transition of HDPCs into the S phase. Furthermore, HDPCs treated with *Stauntonia hexaphylla* extract exhibited a morphologically distinct phenotype characterized by increased elongation and slenderness.

Furthermore, numerous studies have established androgen-induced C57BL/6 mouse models of AGA to validate the in vivo translation of regulatory effects observed in HDPCs via hair recovery rate and alterations in follicular density. For instance, AGA mice treated with *Terminalia bellirica* (Gaertn.) Roxb. crude ethanol extract [117] exhibited skin darkening by day 3 and achieved complete hair regrowth within 14 days, demonstrating a regenerative velocity superior to that of the positive control group treated with finasteride. Moreover, the extract induced a dose-dependent recovery in follicle count by facilitating the transition of hair follicles into the anagen phase. Intriguingly, *Terminalia bellirica* (Gaertn.) Roxb. fruit extract has been demonstrated to mitigate AGA, without influence on murine prostate volume. In contrast, *Connarus semidecandrus* Jack [118] crude ethanol extract exhibits a dual regulatory profile characterized through the inhibition of LNCaP cell proliferation and the concurrent promotion of dermal papilla cell growth, providing a theoretical basis for its potential as an alternative therapeutic modality. Additionally, *Connarus semidecandrus* Jack extract has been confirmed to augment hair shaft thickness in murine models. In vitro experiments performed on isolated murine hair follicles further corroborated the hair growth-promoting efficacy of the *Connarus semidecandrus* Jack ethanol extract.

**Table 3:** Anti-androgenic activity of plant extracts targeting scalp follicle miniaturization and hair growth.

Extract	Plant Part	Extract Type	Chemical Characterization	Research Methods		Key Findings	Ref.
				Model	Dosages Regimen		
*Allium macrostemon* Bunge	Whole dried herb	Crude water extract	UPLC-Q/TOF-MS profiling identified 6 compounds; Adenosine as HPLC quality marker	testosterone-induced alopecia rat model	1% or 3% in 1,2-propanediol, topically sprays, 21 days	Prolong the anagen phase of hair follicles Promoted proliferation and migration Inhibited the apoptosis of HDPCs Regulated the expression of growth factors Activated Wnt/β-catenin pathway	[115]
				HDPCs	0–960 μg/mL		
*Stauntonia hexaphylla*	Leaf	Crude extract	HPLC quality-controlled hederacoside D	androgenetic alopecia mouse model	50 mg/kg, oral, 8 weeks	Improved hair density in alopecic mice Prolongs the anagen phase of hair growth Induced KC proliferation and suppressed apoptosis Reduced the production of DHT Inhibited 5α-R activity Inhibited the androgen signaling	[116]
				HFDPCs	50 μM		
*Connarus semidecandrus* Jack	Whole plant	Crude ethanol extract	GC-MS profiling identification of maltol and DDMP	Testosterone-induced AGA mice model	5 or 10 mg/kg, diluted with PBS and DMSO, 3 weeks	Promoted hair growth and thickening Reduced AR level Inhibitory of 5α-reductase activity Inhibited apoptotic pathway	[118]
				LNCaP and HDP cells	0–400 μg/mL		
*Bacillus*/*Trapa japonica*	Fruit	Fermented water extract filtrate	Lowry assay and HPLC profiling detected six peptide fractions	HDP cells	0.1–1%	Stimulated HDPCs proliferation and migration Induced cell cycle progression Decreased apoptosis Enhanced angiogenesis Inhibited 5α-R1 Activated Akt/ERK/GSK-3β pathway	[119]
				Organotypic 3D cell culture model	1%, for 2 weeks		
*Polygonum multiflorum*	Root	Crude 50% ethanol extract	HPLC analysis of TSG and emodin	HDPCs	10–100 μg/mL	Elongated anagen phase Increased growth factors Abrogated the effect of androgen and prevented hair follicle miniaturization	[120]
*Celtis choseniana* Nakai	Whole plant	Crude 99% methanol extract	HPLC analysis of luteolin	(TP)-induced alopecia rat model	50 or 100 mg/kg, oral, 2 weeks	Increased the hair regrowth rate Promoted telogen-to-anagen transition Reduced the level of DHT Deactivated androgen signaling Promoted cell proliferation via increasing proliferation factor production Modulated Wnt/β-catenin pathway	[56]
				HDPCs	0–200 μg/mL		
*Ipomoea batatas*	Leaf	Crude ethanol extract	LC-MS/MS profiling identified alkaloid compounds and flavonoids	Alopecia rabbit model	1 mL, 21 days	Stimulated hair growth Inhibited the activity of AR Exhibited antifungal activity against *M. furfur*	[34]
*Avicennia marina*	Heartwood	Standardized ethanol extract	NMR and TLC densitometry quantified avicequinone C	DPCs	2.5–20 μg/mL	Induces hair growth at the telogen phase Prevented the apoptosis of DPCs Reduction in the DHT production Stimulate the hair growth factor production Decreased the 5α-R activity Inhibited DHT/T-AR and nuclear translocation	[46]
*Lepidium sativum* L.	Seed	Crude cold-water extract	LC–ESI–MS/MS identified glucosinolates; flavonoid glycosides	Sustanon-induced alopecia rat model	4% in saline, topically sprays, 21 days	Stimulated hair growth factor production Decreased 5α-R in tissues Docking-predicted binding energies with AR ranging from −4.1 to −8.9 kcal/mol	[81]
*Musa* flower	Flower	Crude water extract	HPLC analysis of proanthocyanidins	HFDPCs	0.0625 and 0.125 mg/mL	Increased the hair root diameter and reduced hair loss and scalp redness Increased hair cell growth Decreased the ROS and DHT production Decreased the expression of hair follicle growth inhibition-related genes	[57]
*Justicia procumbens*	Whole plant	Crude water extract	LC-MS/MS and HPLC-DAD identified justicidin A, justicidin B and 4-CQA i	testosterone-induced alopecia B6CBAF1/J mice model	100 or 200 mg/kg, 5 days	Reduced hair loss, increased hair thickness, and enhanced hair shine Alleviated aging-induced epidermal thinning, hair follicle miniaturization Elongated the anagen phase in hair follicles Promoted the proliferation of HFDPC Regulated cell cycle progression Improved vascular health Promotes growth factor production Anti-inflammatory Activated the Wnt/β-catenin pathway	[79,121]
				HFDPCs	0–400 μg/mL		
*Cucumis melo* var. *makuwa*	Leaf	Crude water extract	HPLC–ESI–MS analysis of Meloside A	HDPCs	500–2000 μg/mL	Attenuated DHT-induced HDPCs apoptosis Modulated AR nuclear translocation and gene expression Inhibited ROS production and protects against oxidative stress	[45]
*Angelica* *sinensis* and *Platycladus orientalis*	*Angelica* *sinensis* root and *Platycladus orientalis* leaf	Crude water extract	Reported polysaccharides	C57BL/6 mice model	oral and topical application	Stimulate hair formation Attenuated follicular miniaturization Regulated hair growth factors Inhibition of androgen signaling molecules Anti-inflammatory	[78]
*Mangifera indica*	Leaf	Crude water extract	Reported mangiferin and phenols	Depilated C57BL/6J mice	1%, by applying, 11 days	Promotes hair growth in mice Activated the Wnt signaling pathway Inhibited DKK1 and SRD5A2 expression	[84]
				HFDP Cells	62.5–500 μg/mL		
*Terminalia bellirica* (Gaertn.) Roxb.	Fruit	Crude 50% ethanol extract	Remains to be elucidated	Testosterone-induced alopecia rat model	20 or 100 mg/kg, oral, 2 weeks	Increased hair follicles in the dorsal skin Promoted HFDPC proliferative Induced hair growth via cell cycle Increased hair growth marker expression	[117]
				HDPCs	6.25–100 μg/mL		

The 3D cell models and human hair follicle ex vivo organ cultures provide advanced research platforms with improved physiological relevance for evaluating the anti-androgenetic alopecia activity of plant extracts. *Trapa japonica* fruit [119] extract has been demonstrated to promote human hair follicle dermal papilla cell proliferation via the activation of Akt/ERK/GSK-3β signaling. In this context, an organotypic 3D cell culture model was employed to recapitulate the native hair follicle microenvironment. Similarly, *Polygonum multiflorum* [120] crude ethanol extract has been shown to prolong the anagen phase and delay catagen onset through the upregulation of Bcl-2 and downregulation of DKK-1, in 3D cultured DPC spheroids, which is utilized to mimic the in vivo dermal papilla. Furthermore, in isolated human hair follicle organ cultures, *Polygonum multiflorum* extract has been confirmed to dose-dependently increase the proportion of hair follicles in the anagen phase. This evidence provides further support for the biological activity of *Polygonum multiflorum* extract and highlights its potential relevance for future translational research.

### 4.4. Restoration of Hormonal Balance and Metabolic Homeostasis in PCOS Using Novel Delivery Systems with Plant Extracts

Hyperandrogenism is a major endocrine feature of PCOS. However, PCOS represents a heterogeneous endocrine-metabolic disorder involving hormonal imbalance, metabolic dysfunction, and ovarian abnormalities. Consequently, alterations in the levels of various hormones, including testosterone, LH, follicle-stimulating hormone (FSH), estradiol, and progesterone, serve as critical validation metrics for the efficacy of plant extracts in alleviating PCOS (Table 4). *Nardostachys jatamansi* DC and *Tribulus terrestris* L. crude methanol extract [122], *Matricaria recutita* L. and *Urtica dioica* crude methanol extract [123], and *Ficus religiosa* crude water extract [124] have been demonstrated to significantly reduce testosterone levels, elevate estrogen and progesterone concentrations, and restore the FSH/LH ratio to a physiological range. Notably, the extracts of *Nardostachys jatamansi* DC and *Tribulus terrestris* L. exhibit the most pronounced downregulation of testosterone. Further characterization is needed to determine how individual components contribute to the observed hormonal effects. In terms of reproductive function recovery, all three aforementioned extracts alleviated estrous cycle disorders in PCOS mice. The high-dose *Nardostachys jatamansi* group and fresh *Ficus religiosa* leaf extract group, in particular, exhibited recovery patterns most similar to the physiological state of normal mice. Furthermore, these treatments reduced the number of cystic follicles, increased the counts of developing follicles and corpora lutea, and attenuated ovarian hypertrophy. Interestingly, extracts from *Ficus religiosa*, *Nardostachys jatamansi* DC and *Tribulus terrestris* L., have been found to reverse PCOS-induced uterine atrophy and ameliorate the reduction in endometrial glands and stromal thinning. The observed modulation of hormone levels and histological improvements are primarily attributed to the antioxidant capacity of these plant extracts and their inhibition of the AR.

PCOS is frequently associated with metabolic comorbidities [125,126], including dyslipidemia, obesity, hypertension, and non-alcoholic fatty liver disease. *Saraca asoca* crude ethanol extract [127], *Garcinia cambogia* commercial water extract [27] and *Allium ampeloprasum* crude water extract [128] have been reported to alleviate PCOS-related hormonal imbalance and metabolic alterations in letrozole-induced PCOS rat models. Specifically, the ethanolic extract of *Saraca asoca* not only ameliorated ovarian hormonal dysregulation but also restored hepatic function impaired by PCOS. This effect may be related to various characteristic phytochemical constituents, including kaempferol, rutin, and epicatechin. Conversely, the aqueous extracts from *Garcinia cambogia* and *Allium ampeloprasum* have been demonstrated to target lipid metabolism, significantly reducing levels of total cholesterol and low-density lipoprotein while elevating high-density lipoprotein.

Notably, ethyl acetate fraction of *Coriandrum sativum* [129] extracts and *Foeniculum vulgare* [130] crude extract have introduced novel strategies in pharmaceutical formulation technology. Specifically, microencapsulated *Coriandrum sativum* extract has been demonstrated to significantly ameliorate metabolic, hormonal, and oxidative stress markers in a murine model of PCOS. Similarly, *Foeniculum vulgare* extract exhibits substantial potential in regulating hormonal levels, mitigating oxidative stress, and improving ovarian histomorphology. Moreover, compared to the ethanolic extract, *Foeniculum vulgare* encapsulated in chitosan particles demonstrated superior efficacy in reducing fasting insulin levels and facilitating ovarian restoration. Due to the mucoadhesive properties of chitosan, being anchored on chitosan surface secures the stability of the secondary metabolites of fennel seed extract and prolongs their retention time in the blood stream, leading to enhanced release of the phytomolecules into the target cells. The application of microencapsulation and chitosan-loading technologies enhances the stability and bioavailability of bioactive plant constituents [131], and offers novel insights into targeted drug delivery systems for disease management.

**Table 4:** Anti-androgenic activity of plant extracts targeting polycystic ovarian morphology, ovulatory cycles, and metabolic homeostasis.

Extract	Plant Part	Extract Type	Chemical Characterization	Research Methods		Key Findings	Ref.
				Model	Dosages Regimen		
*Moringa oleifera*	Leaf	Standardized ethanol extract	Proximate and HPLC analysis of flavonoids and phenols	TE-induced PCOS rat model	250 or 500 mg/kg	Reduced oxidative stress Enhanced nephron-hepatic activity	[132]
*Terminalia* *chebula* Retz.	Fruit	Crude ethanol extract	GC–MS characterized of phenolic compounds and fatty acids	letrozole-induced PCOS rat model	100–400 mg/kg, 28 days	Improves the folliculogenesis, ovulation and the uterine cycle Restored the estrous cycle to normal Regulated hormone levels (testosterone↓ LH↓) Regulated estrogen synthesis Reduced oxidative stress (LPX↓)	[62]
*Ficus religiosa*	Leaf	Crude water extract	GC-MS-based characterization of 3-acetoxy 3-hydroxy propionic acid	letrozole-induced PCOS rat model	100 or 300 mg/kg, oral, 30 days	Restored the estrous cycle to normal Regulated testosterone, estrogen, progesterone Inhibited the synthesis of androgen Increased total antioxidant capacity	[124]
*Saraca asoca* (Roxb.) Willd.	Bark	Crude ethanol extract	HPLC of Kaempferol; Rutin, (−)-Epicatechin; Salicylic acid	letrozole-induced PCOS rat model	200–600 mg/kg, gavage, 7 weeks	Reduced body weight and cystic follicles number Regulated hormone levels Decreased oxidative stress and increased antioxidant enzyme levels	[127]
*Garcinia cambogia*	Not specified	Commercial water extract	HPLC, TPC and TFC of phenolic contents; flavonoid contents	letrozole-induced PCOS rat model	100–500 mg/kg, 5 weeks	Reduction in weight, ovarian cysts, improvement of follicle growth Regulated hormone levels (testosterone↓ LH↓) Improvement of insulin sensitivity Increased total antioxidant capacity	[27]
*Coriandrum sativum*	Fresh parts	Ethyl acetate fraction of methanol extract	HPLC and TPC of total phenols	TE-induced PCOS rat model	Remains to be elucidated	Improvement against liver damage Regulated hormone levels (↓: insulin, LH, estradiol; ↑: FSH) Increased antioxidant capacity Anti-inflammatory	[129]
*Foeniculum vulgare*	Seed	Crude ethanol extract; Anchored on Chit-TPP	GC-MS and FTIR analysis of flavonoids and phenolic acids	DHEA-induced PCOS rat model	50 or 150 mg/kg, oral, 15 days	Regulated testosterone, LH and FSH Regulated lipid profile (↓: TC, TG) and glucose Metabolism (↓: insulin) Improved observation of the ovaries Inhibited hypothalamic–pituitary–gonadal axis Decrease the frequency of gonadotropin-releasing hormone release Alleviated the oxidative stress	[130]
*Inonotus obliquus*	Not specified	Commercial ethanol extract	HPLC, TPC, TFC of total phenolic and flavonoid content	letrozole-induced PCOS rat model	100–500 mg/kg	Reduced LH and elevated FSH levels Reduced testosterone and progesterone levels Modulated oxidative stress markers and induced oxidative stress and lipid peroxidation Eliminated ovarian low-grade inflammatory	[67]
*Nothapodytes nimmoniana*	Leaf	Crude methanol extract	GC–MS and HPLC of phytosterols, vitamins D and E	Phytochemical profiling, in silico modeling, and MD simulation		Improved insulin sensitivity Regulated hormonal levels Reduced oxidative stress Anti-inflammatory Targeted TNF-α and hER-α (2AZ5 and 3ERT)	[76]
*Allium ampeloprasum*	Not specified	Crude water extract	Qualitative phytochemical screening, TPC, TTC, TFC	letrozole-induced PCOS rat model	192–768 mg/kg, oral, 15 days	Improved overweight and dyslipidemia Restored the estrous cycle to normal Regulated hormone levels (testosterone↓ LH↓) Reduced oxidative stress	[128]
*Calendula officinalis*	Not specified	Crude 70% ethanol extract	Reported polysaccharides, flavonoids and alkaloids	DHEA-induced PCOS rat model	200–1000 mg/kg	Decreased ovarian tissue damage Regulated testosterone, progesterone and LH Reduced glucose and insulin concentration Improved oxidative stress	[68]
*Cynodon dactylon*	Whole plant	Crude 70% ethanol extract	Reported phenolics and flavonoids	letrozole-induced PCOS rat model	500 mg/kg	Mitigates hormonal imbalances (testosterone↓ estrogen↓ progesterone↑) Improved ovarian and uterine structures Predicted receptor interactions based on molecular docking analysis	[133]
*Matricaria recutita L.* and *Urtica dioica*	Leaf	Crude 50% methanol extract	Reported pigenin, luteolin and quercetin	DHEA-induced PCOS rat model	500 mg/kg Chamomile, 250 mg/kg Urtica dioica, 21 days	Improved folliculogenesis, cystic follicles, and corpus luteum Restored the estrous cycle to normal Phytoestrogenic properties Increased total antioxidant capacity Anti-inflammatory	[123]
*Nardostachys jatamansi* DC, *Tribulus terrestris* L.	Rhizome; Fruit	Crude methanol extract	Remains to be elucidated	EV-treated MC rats Model	5 or 10 mg, intraperitoneal injections, 72 days	Normalized estrous cyclicity Reduced follicular atresia and follicular cysts, and increased the number of corpora lutea Reduced progesterone, testosterone, and estradiol Inhibited the transcriptional activity of the AR	[122]

## 5. Complexity in Anti-Androgenic Efficacy: Mixture Effects and Spatiotemporal Dynamics

Multi-target anti-androgenic effects of plant extracts in diverse androgen-related disorders are inherently shaped by chemical complexity. Unlike purified small-molecule inhibitors, plant extracts are multi-component systems in which efficacy emerges from interactions among coexisting phytochemicals, between distinct plant extracts, and even between plant-derived constituents and clinical drugs. Such interactions may influence anti-androgenic responses by affecting multiple biological pathways and contribute to differences in efficacy and tolerability among plant extracts. They also generate antagonism, metabolic interference, or unstable responses. In parallel, the activity observed under controlled in vitro conditions does not necessarily reflect the actual exposure pattern in vivo. Absorption, biotransformation, tissue selectivity, and temporal changes in constituent ratios continuously reshape the anti-androgenic profile after ingestion [134]. Therefore, understanding plant extracts as dynamic mixtures rather than static agents is essential for accurate evaluation.

### 5.1. Intra-Extract Interactions in Crude Plant Mixtures: Combined and Antagonistic Modulation of Anti-Androgenic Activity

The anti-androgenic activity of plant-derived dietary extracts represents a dynamic system governed by the complex principles of mixture toxicology. A prevailing limitation in current assessments lies in the oversimplification of component interactions, where studies generally focus on demonstrating the anti-androgenic efficacy of a single extract from a holistic perspective. Specifically, there is a lack of adequate consideration regarding the combined effects of multiple bioactive molecules under the models of dose addition and independent action.

Minor bioactive constituents from plant extracts may potentiate the anti-androgenic efficacy of the primary active components through pharmacokinetic or pharmacodynamic interactions [135]. A case in point is *Serenoa repens* extracts used for BPH, whose anti-androgenic mechanism demonstrates typical molecular synergism. Specifically, the free fatty acids are primarily responsible for the direct competitive inhibition of 5α-reductase activity [136]. Concurrently, coexisting phytosterols alleviate the pro-inflammatory microenvironment of prostatic tissue [137,138]. Unsurprisingly, their combined action has been observed to be superior to single components in effectively suppressing androgen-dependent prostate cell proliferation [139].

However, complex phytochemical mixtures do not always imply additive benefits. Instead, they involve intrinsic antagonistic interactions within the extract. The pharmacological activity of certain constituents in crude extracts may counteract the efficacy of primary anti-androgenic components, representing a frequently overlooked toxicological concern in functional food development. Consider *Panax ginseng* extracts, whose intervention on PCa and BPH exhibit complex bidirectional regulation, arising from the functional antagonism among its saponin constituents. Ginsenoside Rg3 acts as a potent anti-androgenic agent that downregulates AR expression, suppresses PSA secretion, and induces apoptosis in PCa cells [140]. Conversely, Ginsenoside Rg1 activates the pro-survival IGF-1/PI3K/Akt pathway and exerts steroid-hormone-mimetic activity and promote cell cycle progression [141]. This divergent regulation results in higher IC_50_ values for growth inhibition by *Panax ginseng* extracts than by purified Rg3 [142], with the net effect largely dependent on the intrinsic Rg3/Rg1 ratio. Such intra-extract antagonism demonstrates that without bioactivity-guided fractionation, the direct use of whole extracts may lead to unsatisfactory therapeutic outcomes due to interference from inactive or antagonistic components.

### 5.2. Inter-Extract Complementarity in Polyherbal Formulations: Potential Functional Integration and Biological Effects

Polyherbal formulation is a standard paradigm in both traditional medicine and modern nutritional science. In contrast to single plant extracts, which prioritize the pharmacological activity of specific constituents, compound botanical formulations establish a multi-component, multi-target, multi-pathway network consistent with network pharmacology through the strategic combination of diverse extracts [143]. For the management of androgen-dependent disorders, the distinct advantage of combinatorial formulations is the systemic modulation of the androgen signaling axis and its downstream microenvironment, which is achieved via the pharmacodynamic complementarity and interactions of distinct phytochemicals, including efficacy enhancement and toxicity mitigation.

KMKKT [102], a composite botanical formulation comprising *Angelica gigas* Nakai and nine other medicinal herbs, exhibits potent anti-androgenic and AR inhibitory activities in androgen-dependent LNCaP prostate cancer. These effects are not merely additive but reflect a synergistic mixture phenomenon that overcomes the therapeutic limitations of AGN extracts or their constituent decursin alone. A critical advantage of the KMKKT is distinct toxicity separation, where the concentration threshold for inducing tumor cell apoptosis is significantly higher than that required for anti-androgenic efficacy. This differs from AGN extracts and decursin, which have a narrower therapeutic window between activity and apoptosis induction. Concurrently, co-formulated herbs traditionally used for systemic support, which contribute anti-inflammatory and antioxidant activities.

A similar principle appears in HX109 [144], in which *Taraxacum officinale* crude extract, *Cuscuta australis*, and *Nelumbo nucifera* are combined to attenuate BPH. The formulation does not simply block AR, but represses AR-responsive transcription through Ca^2+^/calmodulin-dependent protein kinase kinase β (CaMKKβ)-activating transcriptional factor 3 (ATF3) signaling while concurrently reducing prostate hyperplasia in vivo. Thus, mixed extracts can achieve endocrine regulation through pathway convergence instead of high-dose single-target inhibition.

### 5.3. Plant–Drug Combinations: Combined Therapeutic Effects, Metabolic Interference, and Disease Prognosis

Analogous to synergistic regulation among bioactive constituents from plant extracts, combinatorial regimens with clinical therapeutics potentiate anti-androgenic signaling to some extent. However, composition trigger pharmacokinetic antagonism by inducing metabolic enzymes. Flavonoids and terpenoids act as potent ligands for the pregnane X receptor, inducing hepatic and intestinal cytochrome P450 3A4 expression [145]. Because both circulating androgens and many exogenous phyto-antiandrogens are substrates of CYP3A4 [146], such enzyme induction can accelerate metabolic clearance of co-administered agents and reduce systemic exposure. For instance, while *Hypericum perforatum* has been demonstrated to ameliorate inflammation in PCOS [147], its CYP3A4 induction significantly diminishes the plasma concentrations of co-administered clinical antiandrogens, causing impaired efficacy of dietary interventions. Similarly, potent metabolic inducers in crude extracts may compromise the bioavailability of anti-androgenic components, leading to lower overall therapeutic efficacy than purified monomers.

Moreover, the combination of plant extracts and clinical pharmacotherapies facilitates the mitigation of drug-induced adverse effects, offering a new strategy for improving the disorders prognosis. Co-administration of resveratrol and bicalutamide induces a significantly higher apoptotic rate than monotherapy [148], while effectively lowering the required drug concentrations. This dose-reduction strategy retains potent anti-androgenic activity while alleviating high dose-associated adverse events, including hepatotoxicity and fatigue [149]. From a prognostic perspective, ADT is frequently associated with a high risk of metabolic syndrome, including osteoporosis and dyslipidemia [150]. Importantly, plant extracts enhance anti-neoplastic efficacy while preserving systemic homeostasis. For example, soy isoflavone intervention not only exerts mild local anti-androgenic effects to synergistically inhibit tumor proliferation but also mitigates ADT-related bone loss and lipid metabolic disorders via selective estrogen receptor modulation [151,152]. Accordingly, combined treatment with plant extracts and pharmaceutical agents may provide complementary biological effects and could represent a potential strategy for improving supportive care during disease management. However, their effects on quality of life and long-term clinical outcomes require validation in controlled clinical studies.

### 5.4. Spatiotemporal Exposure Dynamics of Plant Extracts: From Static In Vitro Observations to In Vivo Reality

The vast majority of current in vitro assays evaluate the mixture effects at static concentrations, completely neglecting complex spatiotemporal dynamics in vivo. This constitutes a major methodological limitation. Most mixture studies assume fixed ratios and constant concentrations. After oral intake, absorption, phase-II conjugation, microbiota biotransformation, and tissue retention reshape both the chemical identities and the ratios of bioactives that reach androgen-responsive sites [153,154].

Temporally, pharmacokinetic profiles of different constituents within the extract vary dramatically. Taking green tea extracts as an example, although EGCG strongly inhibits 5α-reductase and AR signaling, it possesses an extremely short plasma half-life and is highly prone to oxidation [155]. The analog EC demonstrates higher clearance, while EGCG elimination kinetics are modulated by concomitant compounds [156]. Consequently, the stoichiometric ratio of polyphenols delivered to target organs, such as the prostate or hair follicles, fluctuates dynamically over time after ingestion. Only trace glucuronidated metabolites may remain after several hours, making the EGCG-dominated anti-androgenic efficacy observed in vitro difficult to maintain in vivo.

Spatially, lipophilic phytosterols penetrate the blood–prostate barrier more readily and accumulate in prostatic stroma [157,158], whereas hydrophilic glycosidic components are predominantly restricted to the systemic circulation [159]. This heterogeneous tissue distribution leads to a striking mismatch between actual constituent ratios at target sites and those applied in in vitro systems. Similarly, highly lipophilic bioactives, such as carotenoids or saw palmetto fatty acids, selectively accumulate within the lipid-rich microenvironment of the prostate gland over weeks of continuous intake, achieving local concentrations vastly different from their circulating plasma levels [160,161]. These temporal and spatial discrepancies generate a substantial mismatch between the constituent ratios used in vitro and those actually reaching androgen-responsive tissues in vivo, resulting in a major bottleneck in translating mechanistic research into clinical applications.

## 6. Limitations and Future Perspectives of Anti-Androgenic Plant Extracts: Safety, Research Framework and Application

### 6.1. Identification and Toxicity Evaluation of Anti-Androgenic Bioactive Constituents in Plant Extracts

Plant extracts are chemically complex mixtures. This complexity may contribute to multi-target biological effects but also creates challenges for standardization and safety evaluation. Currently, most studies remain limited to phenotypic screening of crude extracts. Differences in geographical origin, harvest conditions, plant tissues, and extraction procedures may alter phytochemical composition and influence biological activity [162]. Future studies should further standardize extraction procedures and improve chemical characterization to facilitate comparisons of biological activities across different plant extracts. For instance, insufficient standardization of fatty acid composition in *Serenoa repens* extracts lead to marked variability in 5α-reductase inhibitory activity [163]. Therefore, further research is required to combine UHPLC-MS/MS- and NMR-based metabolomics for bioactivity-guided fractionation [164,165,166].

Although generally recognized as safe dietary components with low inherent toxicity, plant extracts still follow the fundamental toxicological principle that biological activity and potential risk are closely dosage-dependent [167]. Consequently, their potential dose-dependent toxic effects cannot be overlooked. In response to growing concerns over the quality and safety, recent efforts at the National Toxicology Program have focused on the toxicity of anti-androgenic plant-derived dietary supplements [168], including green tea and *Panax ginseng*. Reproductive toxicity and endocrine disruption are primary concerns when evaluating potential toxicological mechanisms of plant extracts [169]. Hepatotoxicity and nephrotoxicity also represent critical hazards that cannot be neglected [170]. Currently, there is a significant gap regarding matched pharmacokinetic, dynamic, and toxicological data for plant extracts. This data deficiency severely limits the statistical correlation between static in vitro observations and dynamic in vivo evidence. Comprehensive evaluation of absorption, distribution, metabolism, excretion, and toxicity is therefore essential during the development of anti-androgenic dietary supplements. These procedures include the construction of dose–response curves, the identification of No-Observed-Adverse-Effect Level, and targeted safety evaluation under long-term chronic exposure [171].

### 6.2. Establishment of a Multidimensional System for Evaluating the Anti-Androgenic Plant Extracts

#### 6.2.1. Fundamental In Vitro and In Vivo Models for Validating the Anti-Androgenic Extracts

**Figure 3:**
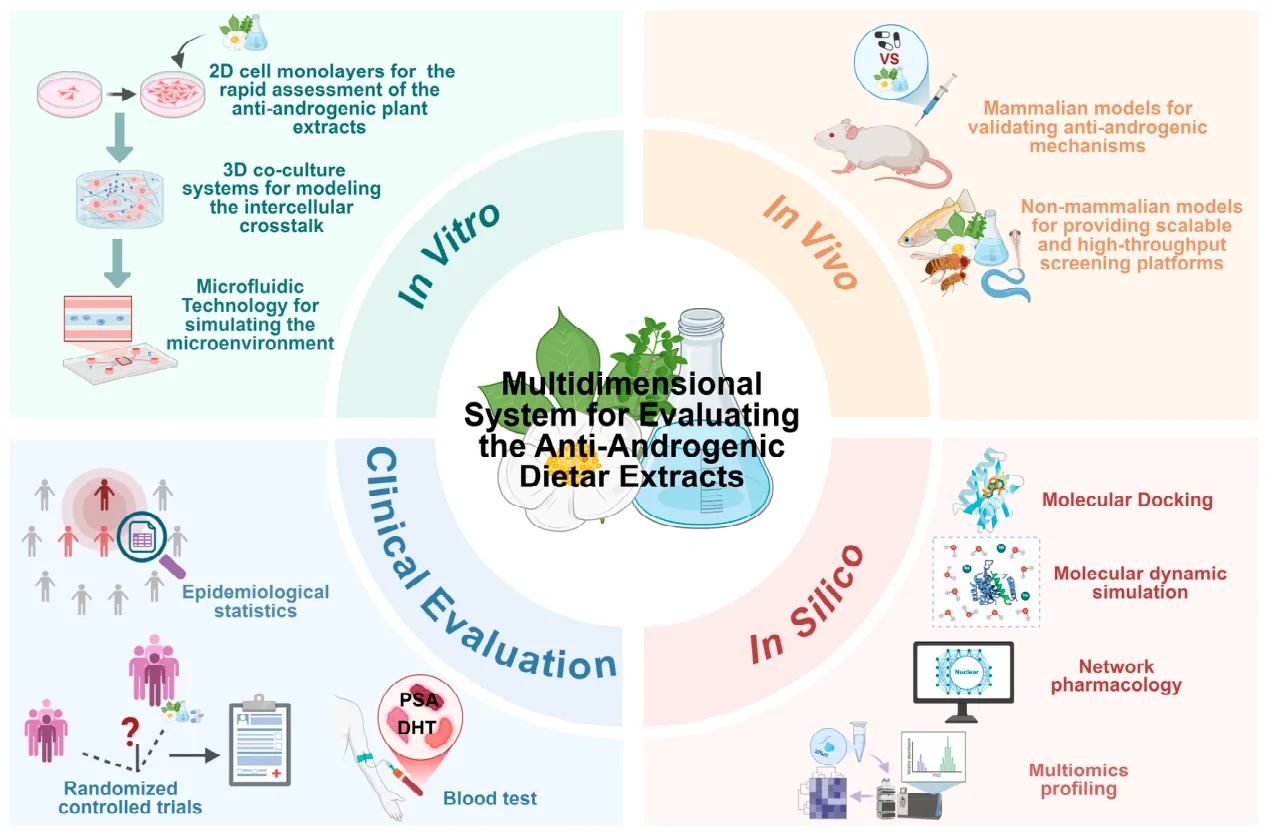
Multidimensional system for evaluating the anti-androgenic plant extracts. There are four complementary domains, including in vitro screening, in vivo validation, in silico target modeling, and epidemiological and clinical assessment. Created with biorender.com.

Elucidating the anti-androgenic mechanisms of plant extracts requires a multitiered experimental framework (Figure 3). These models, spanning from simplified cell cultures to complex animal systems, offer a comprehensive investigative platform. They are indispensable for progressing beyond preliminary activity screening to establish mechanistic causality and define dose–response relationships. This integrated strategy is pivotal for overcoming the inherent limitations associated with reliance on single experimental models. The following sections critically evaluate the applications, advantages, and limitations of these platforms, which collectively generate the foundational data needed to develop predictive models and support evidence-based nutritional interventions.

In vitro models serve as a cornerstone for mechanistic investigation, offering critical insights into the responses of specific cell types to endocrine hormonal regulation. Conventional 2D cell monolayers serve as fundamental screening platforms for the rapid assessment of the anti-androgenic plant extracts. BPH cell models [172], consist of DHT-induced human normal prostate epithelial and stromal cells, have been widely applied to assess the cytotoxicity of *Psoralea corylifolia* L. [112,173] crude extracts. Moreover, 3D scaffold-free co-culture systems integrating prostate epithelial and stromal cells are essential for modeling the intricate intercellular crosstalk [174]. By capturing cellular heterogeneity and microenvironmental interactions, these models dissect indirect anti-androgenic effects of plant extracts, including the attenuation of androgen-dependent epithelial proliferation via stromal-derived pro-proliferative factors suppression. Nevertheless, even advanced 3D co-culture systems are limited in replicating the complex in vivo metabolic clearance and immune surveillance networks. Microfluidic technologies are a frontier in this field [175]. Prostate-on-a-chip models integrate fluid shear stress and dynamic hormonal fluctuations to simulate the prostate microenvironment [176]. Despite the growing use of such systems in prostate cancer diagnosis and treatment, screening anti-androgenic components remains a promising and underexplored area.

In vivo models are indispensable for validating biological activity. Rodents feature high physiological relevance with a conserved hypothalamic–pituitary–gonadal axis and complete metabolic clearance system [177]. These models are critical for linking long-term plant extract exposure to reproductive and developmental outcomes. The conventional Hershberger assay is the gold standard for quantifying in vivo anti-androgenic potency by measuring weight alterations in androgen-dependent tissues [178]. In contrast, non-mammalian models provide scalable and high-throughput screening platforms. The zebrafish model straightforward evaluates chemical-induced androgen receptor signaling blockade through phenotypic alterations, including impaired courtship behavior, reduced sperm motility, and decreased offspring survival rate [179]. Furthermore, transgenic fluorescent zebrafish lines permit non-invasive visualization of AR activity in vivo [180]. However, substantial physiological differences exist between mammalian and non-mammalian systems. Owing to the absence of specific androgen target organs, models including zebrafish, *Caenorhabditis elegans* [181,182], and Drosophila [183,184] are mainly suitable for high-throughput screening of anti-androgenic plant extracts. The detailed mechanisms focused on androgen-related disorders still requires validation in mammalian models.

#### 6.2.2. Multiomics Profiling and In Silico Models for Target Simulation and Network Construction

While traditional in vitro and in vivo models have preliminarily validated the anti-androgenic activity of plant extracts, resolving the underlying molecular mechanisms—particularly multi-component interactions in complex matrices, low-dose chronic exposure effects, and target specificity—requires integration of advanced multiomics profiling and in silico models. Methodologies including molecular docking, network pharmacology, and multiomics integration are shifting the research paradigm from phenotypic observation alone to the elucidation of molecular network regulation [185,186]. These tools are essential for constructing a multidimensional research framework that guides precise screening of bioactive constituents, reveals crosstalk between androgen receptor signaling and metabolic pathways, and establishes a predictive basis for structure–activity relationship analysis.

Molecular docking generates hypotheses about plant extract occupation of the AR LBD by predicting binding affinities and spatial conformations [187]. However, conventional docking is limited by its static nature, which fails to capture dynamic conformational changes in the receptor after ligand binding. Specifically, AR antagonistic activity often depends on Helix-12 conformational displacement, which static models cannot accurately predict [188]. To address these limitations, molecular dynamics simulations [189] are becoming mainstream. These simulations assess receptor–ligand complex stability in a physiological environment over nanosecond timescales, reveal allosteric modulation by dietary constituents and distinct between true antagonists and potential agonists. Furthermore, to elucidate the multi-component-multi-target synergistic mechanisms of plant extracts, network pharmacology [190] provides a systems-level analytical. This approach identifies key driver active ingredients in complex matrices and predicts their synergistic enhancement of anti-androgenic efficacy via modulation of inflammatory or apoptotic pathways.

To transition from predictive modeling to empirical validation, multiomics profiling including transcriptomics, proteomics, and metabolomics provide an unbiased systems-level view of cellular responses to dietary interventions. By integrating these high-dimensional datasets, researchers establish data-driven profiles of anti-androgenic activity, and identify critical regulatory nodes that may be neglected in traditional hypothesis-driven studies. This framework supports comprehensive analysis of anti-androgenic molecular cascades triggered by dietary interventions. Furthermore, spatial metabolomics offers unprecedented spatiotemporal resolution [191]. Notably, metabolites can serve as sensitive biomarkers for the early evaluation of anti-androgenic effects induced by dietary interventions, with their alterations occurring much earlier than histopathological changes.

#### 6.2.3. Integrated Epidemiological and Clinical Evaluation System for Precise Anti-Androgenic Intervention

Current evidence for anti-androgenic plant extracts is mainly derived from preclinical studies, observational investigations, and clinical intervention trials. However, these evidence types remain uneven. Mechanistic evidence from experimental models is substantially greater than human evidence. In vitro, in vivo, and in silico studies have provided important insights into the biological activities and potential mechanisms of plant extracts. Epidemiological studies and clinical interventions are needed to determine their effects in human populations.

Observational studies are a critical bridge between in vitro molecular mechanisms and population-level dietary effects. Recent investigations have linked dietary patterns to changes in reproductive hormone levels. For example, diets rich in genistein show statistical correlations with reduced semen quality parameters or altered reproductive hormone profiles [192]. These findings implicate the potential cumulative effects of dietary anti-androgenic exposures at the population level [193]. However, these associations are often masked by confounding factors such as lifestyle, genetic background, and co-exposures. Consequently, cross-sectional or retrospective surveys alone are insufficient for establishing definitive causal relationships. The integration of biomarker monitoring may further improve the accuracy of exposure assessment.

Compared with observational evidence, clinical intervention studies, particularly randomized controlled trials (RCTs), provide stronger evidence for evaluating causal effects of plant-derived interventions. However, despite promising anti-androgenic efficacy demonstrated in preclinical models, clinical evidence for plant extract mixtures remains limited. Over the past decade, RCTs for PCa, BPH, AGA, and PCOS have predominantly focused on isolated phytochemicals rather than complex botanical mixtures. Furthermore, the clinical evidence for mixed extracts is largely confined to the application of *Serenoa repens* in prostate and hair disorders. The absence of rigorous RCTs for plant extract mixtures represents a major gap in current clinical evidence. Existing clinical interventions primarily assess biological responses using androgen-related surrogate biomarkers, such as PSA kinetics and serum testosterone levels in PCa and BPH populations [194,195]. These metrics provide clinically relevant surrogate measures for evaluating potential anti-androgenic effects. Recent high-quality meta-analyses have clarified the complex regulatory profiles of plant extracts [196]. These findings highlight the critical role of dose–response dynamics, exposure windows and baseline hormonal status in determining clinical outcomes. Many intervention studies lack sufficient statistical power due to small sample sizes or short intervention durations. Additionally, metabolic heterogeneity in individual gut microbiota may mask true biological effects, leading to significant inter-individual variability in clinical responsiveness [197,198].

A key future advancement is the development of an integrated evaluation framework to bridge basic research and clinical application. This holistic approach corrects biases in single-model predictions and provides rigorous scientific logic to establish causal anti-androgenic mechanisms, advancing the field from empirical dietary supplementation to mechanism-based precision nutrition [199].

### 6.3. Improving Stability and Bioavailability of Dietary Extracts via Advanced Technologies

Plant extracts have attracted considerable interest as potential candidates for functional foods and dietary interventions targeting androgen-related disorders [200]. This potential is supported by their reported effects on androgen-related pathways. However, a considerable gap remains between bioactivity in experimental models and their translational relevance under physiological conditions. Limited chemical stability, variable composition, and insufficient bioavailability may influence the exposure and biological availability of active constituents.

Methodological limitations in extraction can affect the stability and recovery of anti-androgenic constituents [201,202]. Solvent-based thermal reflux efficiently disrupts the cell wall but often induces sterol side-chain oxidation and double-bond isomerization due to prolonged exposure to high temperatures and pressures [203,204]. In contrast, organic solvent cold maceration alleviates thermal degradation but exhibits low dissolution efficiency for intracellularly bound components, resulting in reduced target yields and compromised bioactivity [205]. Emerging supercritical CO_2_ extraction permits non-destructive processing at low temperatures but displays an inherent extraction gap for highly polar flavonoid glycosides [206]. Extraction inherently involves the disruption of plant cell walls and native colloidal systems, which enriches target compounds but eliminates their endogenous antioxidant defenses. Loss of this natural matrix exposes free bioactive molecules to environmental stresses, rendering them vulnerable to physicochemical alterations. Future technological development should prioritize integrated extraction-encapsulation systems [207]. Spray drying or microencapsulation performed immediately after extraction forms molecular cages with cyclodextrins or modified starches to effectively protect them against photo-oxidative and thermal degradation [208,209].

Improving the exposure of bioactive constituents in target tissues, such as the prostate and scalp, may enhance the anti-androgenic effects of plant extracts. During gastrointestinal digestion and metabolic transformation, active compounds may undergo degradation or reduced absorption, limiting their effective exposure in vivo. Therefore, the development of novel nano-delivery systems is a key strategy to promote the clinical translation [210,211]. In addition, functional excipients [212] provide complementary strategies to improve the stability, retention, and bioavailability of anti-androgenic constituents in plant supplements.

## 7. Conclusions

In this review, we summarized the crosstalk network underlying the anti-androgenic effects of plant extracts, particularly those from dietary sources. Up to now, plant extracts have been shown not to target a single isolated molecular event, but to act at multiple nodes of the AR signaling axis, including antagonism at the ligand-binding domain, suppression of AR expression, reduced AR protein stability, restriction of AR nuclear translocation, and inhibition of AR splice variants. Plant extracts reduce androgen levels by inhibiting key steroidogenic enzymes that regulate intracellular androgen synthesis. More importantly, these effects are embedded within broader signaling crosstalk, including cell proliferation and apoptosis, inflammation, oxidative stress and growth factor regulation. This indicates that the anti-androgenic potential of plant extracts should be understood not as a simple extension of conventional androgen blockade, but as a systems-level regulatory paradigm. This paradigm operates across androgens synthesis, AR signaling, and downstream androgen dysregulation associated pathological remodeling. Such mechanistic network may partly explain their potential relevance across distinct androgen-related disorders, particularly in contexts involving long-term intervention, multi-target regulation, or resistance-associated mechanisms.

Moreover, the multi-component mixtures present in crude plant extracts, the varying ratios of different plant extracts in botanical formulations, and the combination of plant extracts with pharmaceutical compounds drive synergistic or antagonistic interactions among bioactive molecules, resulting in dual regulation of anti-androgenic activity. Meanwhile, spatiotemporal dynamics arising from differences in metabolic biotransformation, exposure kinetics, and tissue specificity further complicate the anti-androgenic activity and its dose- and time-dependency. This constitutes the fundamental challenge in translating plant extracts from mechanistic research to clinical applications.

Addressing these challenges will require a paradigm shift toward integrated research framework. Future studies should focus on the concrete directions: first, establish standardized reference extracts via bioactivity-guided fractionation; second, conduct rigorous, large-scale RCTs for PCa, AGA, and PCOS; third, develop AR-V7-targeting plant formulations via advanced nano-delivery platforms. Ultimately, this research framework facilitates the transition of plant extracts from empirical nutritional supplements to standardized, precisely targeted botanical interventions, realizing their potential for the safe and long-term management of androgen-driven disorders.

## Data Availability

No new data were created or analyzed in this study. Data sharing is not applicable to this article.

## Abbreviations

The following abbreviations are used in this manuscript:

17β-HSD	17β-hydroxysteroid dehydrogenase
2D	Two-dimensional
3D	Three-dimensional
ADT	Androgen deprivation therapy
AGA	Androgenetic alopecia
AR	Androgen receptor
AREs	Androgen response elements
AR-FL	Full-length androgen receptor
AR-Vs	Androgen receptor variants
ATF 3	Activating transcriptional factor 3
Akt	Protein kinase B
Bcl-2	B-cell lymphoma-2
BPH	Benign prostatic hyperplasia
CaMKKβ	Calmodulin-dependent protein kinase kinase β
CDK4	cyclin D-dependent kinase 4
COX-2	Cyclooxygenase-2
CRPC	Castration-resistant prostate cancer
CTGF	Connective tissue growth factor
CYP17A1	Cytochrome P450 17A1
CYP3A4	Cytochrome P450 3A4
c-Myc	Cellular myelocytomatosis oncogene
DHEA	Dehydroepiandrosterone
DHT	Dihydrotestosterone
DP	Dermal papilla
DKK1	Dickkopf WNT signaling pathway inhibitor 1
EC	Epicatechin
EGCG	Epigallocatechin gallate
ERK	Extracellular signal-regulated kinases
FASN	Fatty acid synthase
FGF	Fibroblast growth factor
FGF-7/10	Fibroblast growth factor 7/10
FSH	Follicle-stimulating hormone
GnRH	Gonadotropin-releasing hormone
GSK-3β	Glycogen synthase kinase-3β
H3K27	Histone H3 lysine 27
HDPCs	Human dermal papilla cells
HER2/3	Human epidermal growth factor receptor 2/3
HPO	Hypothalamic–pituitary–ovarian axis
IL-6	Interleukin-6
IL-8	Interleukin-8
IL-1β	Interleukin-1β
iNOS	Inducible nitric oxide synthase
IGF-1	Insulin-like growth factor 1
JNK	c-Jun N-terminal kinase
LBD	Ligand-binding domain
LH	Luteinizing hormone
LHR	Luteinizing hormone receptor
MAPK	Mitogen-activated protein kinase
mTOR	Mammalian target of rapamycin
mRNA	Messenger ribonucleic acid
NF-κB	Nuclear factor-kappa B
NMR	Nuclear magnetic resonance
p38	p38 mitogen-activated protein kinase
PCa	Prostate cancer
PCOS	Polycystic ovary syndrome
PKA	Protein kinase A
PI3K	Phosphatidylinositol 3-kinase
PSA	Prostate-specific antigen
ROS	Reactive oxygen species
SMYD3	SET and MYND domain-containing protein 3
SRC-1	Sterol receptor coactivator-1
SREBP-1	Sterol regulatory element-binding protein 1
SRD5A	Steroid 5α-reductase
SRD5A1	Steroid 5α-reductase isoform 1
SRD5A2	Steroid 5α-reductase isoform 2
SRD5A3	Steroid 5α-reductase isoform 3
TGF-β1	Transforming growth factor-beta 1
TNF-α	Tumor necrosis factor-α
VEGF	Vascular endothelial growth factor
